# Exploring the microbial community (microflora) associated with ovine *Haemonchus contortus* (macroflora) field strains

**DOI:** 10.1038/s41598-017-00171-2

**Published:** 2017-03-06

**Authors:** Saeed El-Ashram, Xun Suo

**Affiliations:** 1 State Key Laboratory for Agrobiotechnology & College of Veterinary Medicine, China Agricultural University, Beijing, 100193 China; 2 National Animal Protozoa Laboratory & College of Veterinary Medicine, China Agricultural University, Beijing, 100193 China; 3 Key Laboratory of Animal Epidemiology and Zoonosis of Ministry of Agriculture, Beijing, 100193 China; 4 Faculty of Science, Kafr El-Sheikh University, Kafr El-Sheikh, Egypt

## Abstract

High-throughput sequencing technology has shown tremendous promise for microbial community composition and diversity. Illumina MiSeq platform was exploited to study the microbial community associated with the different stages of the life-cycle of ovine *Haemonchus contortus* field strains using two distinct amplification primer sets (targeting V3–V4, and V5–V7). Scanning electron microscope and polymerase chain reaction coupled with Illumina MiSeq platform were employed to confirm the absence of any parasite surface contamination by intact bacteria or their DNA products. Results showed 48 (V3–V4 tags) and 28 (V5–V7 tags) bacterial genera comprised the microbial flora of *H. contortus* microbiome. The dominant bacterial genera belonged to *Escherichia-Shigella*, *Pseudomonas* and *Ochrobactrum,* which were shared in all the stages of the parasite life-cycle using V3–V4 and V5–V7 amplicons. Moreover, the parasite microbiome could reflect the external micro-organisms (i.e. micro- and macro-habitats). There is abundant room for further progress in comparing microbiome of different helminths, which has, and will continue to offer considerable potential for better understanding a wide-variety of devastating animal and human diseases.

## Introduction

Among the gastrointestinal parasites that cause losses to the farming industry, for example, *Haemonchus, Ostertagia, Trichostrongylus*, *Nematodirus* and *Cooperia*, the barber’s pole worm *Haemonchus contortus* is the predominant, blood- sucking, highly pathogenic, and economically important nematode that infects small ruminants. It has been demonstrated that larvae provoked tiny haemorrhages as early as 3-day post-infection (dpi). Emergence of the larvae into the abomasal lumen commenced between 7- and 11-dpi and all worms had moulted to the 4^th^ stage by 4-dpi. The early 4^th^ stage (L_4_) has a provisional buccal capsule, which facilitated larvae to attach to the abomasal mucosa and suck blood for the first time^1^. All mammals harbor a wide diversity of microbes that live in harmony with their host and colonize the mucosal surfaces, including digestive tract, respiratory surfaces and reproductive tract^2^. Our knowledge of the symbiosis between bacteria and eukaryotes has grown rapidly in recent years. It has conclusively been shown that the α-proteobacterium *Wolbachia pipientis* causes reproductive manipulation of the arthropod and filarial nematode hosts in various ways, including feminization, parthenogenesis, male killing and sperm-egg incompatibility^3,4^. Apart from the well-studied nematode-associated *Wolbachia*, there appear to be few reports of studies designed to identify the bacteria associated with *Ascaris suum* ^5,6^ and *Trichuri muris* ^7^. More recent attention has focused on the alteration of nematode entomopathogenicity by *Rhabditis regina* microbiome^8^. The hypervariable regions of 16S ribosomal RNA (rRNA) provide species-specific signature sequences. The Polymerase Chain Reaction (PCR)-amplified bacterial 16S rRNA gene sequencing has been emerged as the backbone of microbial community studies over recent decades owing to major breakthroughs in nucleic acids sequencing technology (the development of Next-Generation Sequencing technology) and molecular techniques^9^. DNA sequencing of 16S rRNA genes or gene fragments has played a key role in large ongoing microbial community studies, such as the National Institutes of Health (NIH) funded human microbiome project^10^, human and animal microbiome studies during parasitic infection^11,12^ to index the bacterial communities in a sample without the bias or cultivation effort. Currently, this is the most reliable approach to explore the diversity of microbial community and to determine its composition. We employed 16S rRNA gene amplification and Illumina MiSeq platform to portray, for the first time to our knowledge, the differences in composition and relative abundance of diverse *H. contortous* microbiome. This study provides new insights into the microbial community naturally associated with field strains of *H. contortus* life-cycle stages, with the long-term novel approach of manipulating them to control ruminant gastrointestinal parasites.

## Materials and Methods

### Ethics approval and consent to participate

Animal experiments were conducted in accordance with the guidelines of Beijing the Municipality on the Review of Welfare and Ethics of Laboratory Animals approved by the Beijing Municipality Administration Office of Laboratory Animals (BAOLA), and under the protocol (CAU-AEC-2010–0603) approved by the China Agricultural University Animal Ethics Committee. All experimental procedures were also approved by the Institutional Animal Care and Committee of China Agricultural University (The certificate of Beijing Laboratory Animal employee, ID: 15883).

### Animals and parasites

Field microscopic investigation on *H. contortus* in 107 sheep in an animal farm in Jin Zhan, Cun, Chaoyang, Beijing, China to determine whether any general characteristics of the sheep were associated with infections were performed to select *H. contortus*-infected animals for the current study. Parasitological examination of rectal fecal samples from the sheep was analyzed with the modified McMaster technique^13^. In addition, FAMACHA^©^ scores were performed for detecting anemic sheep on the farm by matching the color of the ocular conjunctival mucous membranes of each animal to the FAMACHA^©^ eye color chart, with the scale ranging from 1 to 5, where a score of 1 is valued not anemic and 5 is valued highly anemic^14^. Body condition scoring (BCS) was assessed by observing and palpating the loin area (behind the ribs and in front of the pelvis), and assigning a score of 1–10 according to the procedure used by^15^ with 1 being emaciated and 10 being extremely fat. Blood was collected for haemato-biochemical to reconfirm the presence of *H. contortous* infection. The aforementioned parasitological tests, FAMACHA^©^ scores and body condition scoring and haemato-biochemical analyses were reassessed after two weeks to ensure the consistency of the results.

A total of three out of fourteen *H. contortus*-infected sheep; two-month-old sheep (*Ovis aries*) that were randomly selected and brought from the local farmer in Jin Zhan village, Chaoyang, Beijing, China. Upon arrival in China Agricultural University facilities, all sheep were tagged (each sheep was assigned an arbitrary number for sample identification purposes). Feces from the infected sheep were collected and cultured at room temperature for 7 days to allow the third stage larvae (the infectious stage) to develop from the eggs, and larvae were harvested using the Baermann technique^16^. The remaining feces together with the abomasal contents were collected from *H. contortus*-infected sheep immediately after death, and eggs were extracted employing sucrose step-density gradient centrifugation (unpublished data). The collected eggs were washed on a 35 µm sieve with distilled water to remove the sugar under sterile conditions. The mesh sieve is present at the top of ceramic funnel and the flask with one side-arm attached to rubber tube connected with another double-side arm flask. The latter is connected with vacuum pump, which has an advantage over the ordinary gravitational force. The adult worms were recovered from the abomasal mucosa using forceps and dissecting microscope. Before the DNA was extracted *H. contortus* adult worm, exsheathed L_3_ and eggs were washed with phosphate-buffered saline (PBS, pH 7.4) and incubated in an antibiotic solution (1 mg/ml ampicillin/1 mg/ml gentamicin) for 2 h to kill external bacteria. Then, the parasites were washed twice with 2% sodium hypochlorite for 20 s each followed by five times with sterile PBS.

### Ruling out bacterial contamination associated with the parasite surfaces

Several tests, such as scanning electron microscope (SEM) and polymerase chain reaction (PCR) were conducted to rule out any abomasal microbial contamination associated with the surface of the distinct life-cycle stages of *H. contortus*.

**Figure 1:**
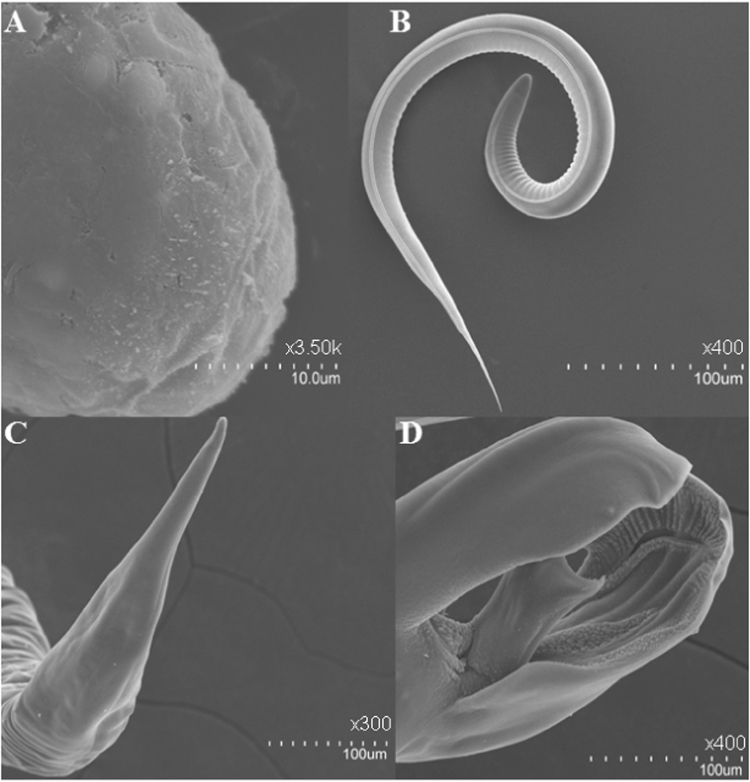
Transmission electron microscope (TEM) images of the different life -cycle stages of *H. contortus*. (**A**) *H. contortus* egg. (**B**) *H. contortus* larva. (**C**) *H. contortus* adult female worm (posterior end). (**D**) *H. contortus* adult male worm (posterior end).

Parasites were prepared for SEM as previously reported by^17^ and Hitachi S-3400N SEM was employed to assure the absence of any surface bacteria on the selected life-cycle stages of *H. contortus* (Fig. 1). So in addition to SEM, PCR further confirmed the absence of bacterial contamination from the abomasal microbial community. The approach to distinguishing between unwashed and washed individual female worms, surface washes, which were washed following the procedure above, involved PCR and next-generation sequencing. Parasites were stored at −20 °C for downstream applications. The procedures launched are described in detail in the Materials and Methods section below.

### DNA extraction, amplification and processing of samples for high-throughput sequencing

#### Microbial DNA extraction

*H. contortus* genomic DNA together with microbial genomic DNA from samples was extracted using the E.Z.N.A.® Bacterial DNA Kit (Omega Bio-tek, Norcross, GA, US) according to manufacturer’s instruction. DNA products were assessed by electrophoresis in 1% agarose gel run at 100 V for 30 min, and the sizes of the products (23 kb) were validated by comparison with a molecular size standard. The quantity and quality of the DNA were evaluated by measuring the absorbance employing an ultraviolet spectrophotometer (DU1 640, Beckman Instruments, Inc., CA, USA) at 260 and 280 nm. All microbial genomic DNA was stored at −20 °C before further analysis.

#### Amplicon generation and high-throughput sequencing

**Figure 2:**
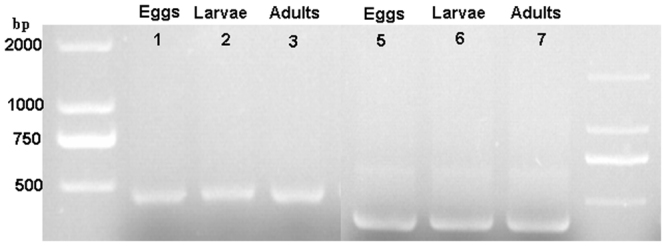
GoldViewTM Nucleic Acid Stained 1.5% Agarose gel of V3-V4 region of 16S RNA gene sequence (1, 2 and 3) and V5-V7 region of 16S RNA gene (4, 5 and 6) amplified product of eggs, L_3_ larvae and adults of *H. contortus.* Lane 1 and 8: Direct-load™ Star Marker Plus (D2000 Plus); GenStar Biosolutions Co. Ltd, Beijing, China.

The 16S ribosomal RNA (rRNA) gene contains of nine hypervariable regions enclosed by regions of more conserved sequence. As shown in Fig. 2, regions of approximately 468 bp and 394 bp encircling the V3-V4 and V5-V7 hypervariable regions respectively within the 16S rRNA gene was subjected to high-throughput sequencing to maximize the effective length of sequencing reads^18^.

Sequencing was done on the Illumina MiSeq® platform at Beijing Allwegene Technology Co., Ltd, China. The V3-V4 and V5-V7 regions of the bacterial 16S rRNA gene sequences were amplified employing a set of commonly used primers for the analysis of *H. contortus*-associated bacterial communities using Illumina MiSeq platform (Table 1).

**Table 1:** Summary of primers used in the current study.

Primer pairs	Primer sequence (5′−3′)	Hypervariable region of the 16S rRNA gene sequence targeted by primer pairs.	References
338F	5′-ACTCCTACGGGAGGCAGCAG-3′	V3-V4 region	Wu, X. *et al*.^18^
806R	5′-GGACTACHVGGGTWTCTAAT-3′		
799F	AACMGGATTAGATACCCKG	V5-V6-V7 region	Beckers *et al*.^32^
1193R	ACGTCATCCCCACCTTCC		

Briefly, each 50 μL of PCR reaction contains 10 ng of abomasal or ruminal microbial genomic DNA as the template, 0.25 μL Pyrobest™ DNA Polymerase (Takara Biotechnology, Dalian, CO., LTD) and 1 μL of 10 μM of each primer. PCR reactions were carried out using the following protocol: (1) an initial denaturation step performed at 95 °C for 5 min followed by 30 cycles of denaturation (95 °C, 30 s), annealing (56 °C, 30 s) and extension (72 °C, 40 s), and a final elongation of 10 min at 72 °C. The PCR products were separated by 1% agarose gel electrophoresis, and the 468 bp and 394 bp fragments were purified by using E.Z.N.A.® Gel Extraction kit (Omega Bio-tek, GA, US). Sequencing libraries were generated using NEBNext^®^ Ultra DNA Library Prep Kit for Illumina^®^ (Illumina, USA) following manufacturer’s recommendations and index codes were added. The library quality was assessed on the Qubit@ 2.0 Fluorometer (Thermo Scientific) and Agilent Bioanalyzer 2100 system. Then, the library was sequenced on an Illumina MiSeq platform (Beijing Allwegene Technology Co., Ltd, China) and 300 bp paired-end reads were generated.

#### Statistical and bioinformatics analysis

After trimming the adaptor and primer sequences from Illumina reads, the raw sequences were assembled for each sample according to the unique barcode using Qiime (V1.7.0, http://qiime.org/scripts/split_libraries_fastq.html).

Paired-end reads from the original DNA fragments were merged using FLASH (V1.2.7, http://ccb.jhu.edu/software/FLASH/).

Quality filtering was performed under specific filtering conditions to obtain the high-quality clean tags according to the pipeline tool quantitative insights into microbial ecology Qiime (V1.7.0, http://qiime.org/scripts/split_libraries_fastq.html).

The clean tags were compared against the reference database (Gold database, http://drive5.com/uchime/uchime_download.html) employing UCHIME algorithm (UCHIME Algorithm, http://www.drive5.com/usearch/manual/uchime_algo.html) to detect and remove chimeric sequences. Paired-end reads were assigned to each sample according to the unique barcodes. Sequence analysis was performed by UPARSE software package (Uparse v7.0.1001, http://drive5.com/uparse/) using the UPARSE-OTU and UPARSE-OTU ref algorithms. Alpha (within samples) and beta (among samples) diversities were analyzed employing in-house Perl scripts. Sequences with ≥97% identities were assigned to the same OTUs. Representative sequences for each OTU were picked up to be used in the ribosomal database project (RDP) classifier to annotate the taxonomic information for each representative sequence. In order to estimate alpha diversity, we rarified the operational taxonomic units (OTUs) and calculated five metrics: Phylogenetic diversity (PD) Whole tree, Good’s coverage, Chao1, Shannon and Observed species. Rarefaction curves were created based on this five metrics.

The Kruskal-Wallis test^19^ was used to compare diversity metrics, such as genus evenness, Simpson and Shannon-Wiener species diversity indices. The level of significance was determined at *P* < 0.05.

The Principal Coordinate Analysis (PCoA) was conducted to explore the differences in the bacterial community structures and was displayed with the WGCNA, stat and ggplot2 packages in the R software (Version 2.15.3). Weighted and un-weighted unifrac distances were calculated employing QIIME^20^. Graphical representation of the relative abundance of bacterial diversity from phylum to genus was visualized using a bar plot with horizontal bars.

## Results

### Parasitological tests, FAMACHA^©^ scores and body condition scoring

For coprological examinations, the rectal fecal samples were collected for fecal egg count examination employing the modified McMaster technique. *H. contortus* were found in 14 (13.08%) of the 107 sheep studied. Additionally, FAMACHA^©^ scores and body condition scoring for all 14 infected animals were assessed. Our results showed that FAMACHA^©^ scores of all 14 infected animals fell between 2 and 3 out of 5 and no sheep decreased below 6 or increased above 8 of all 14 infected sheep for body condition scores. Blood samples were collected for haemato-biochemical analyses to confirm the presence of *H. contortus* infection. Interestingly, these results further support the coprologic examinations (unpublished data). The aforementioned parasitological tests, FAMACHA^©^ scores, body condition scoring and haemato-biochemical analyses were reassessed after two weeks; the results are consistent with the previously obtained data.

### Morphological forms of the ovine *H. contortus* life-cycle stages

**Figure 3:**
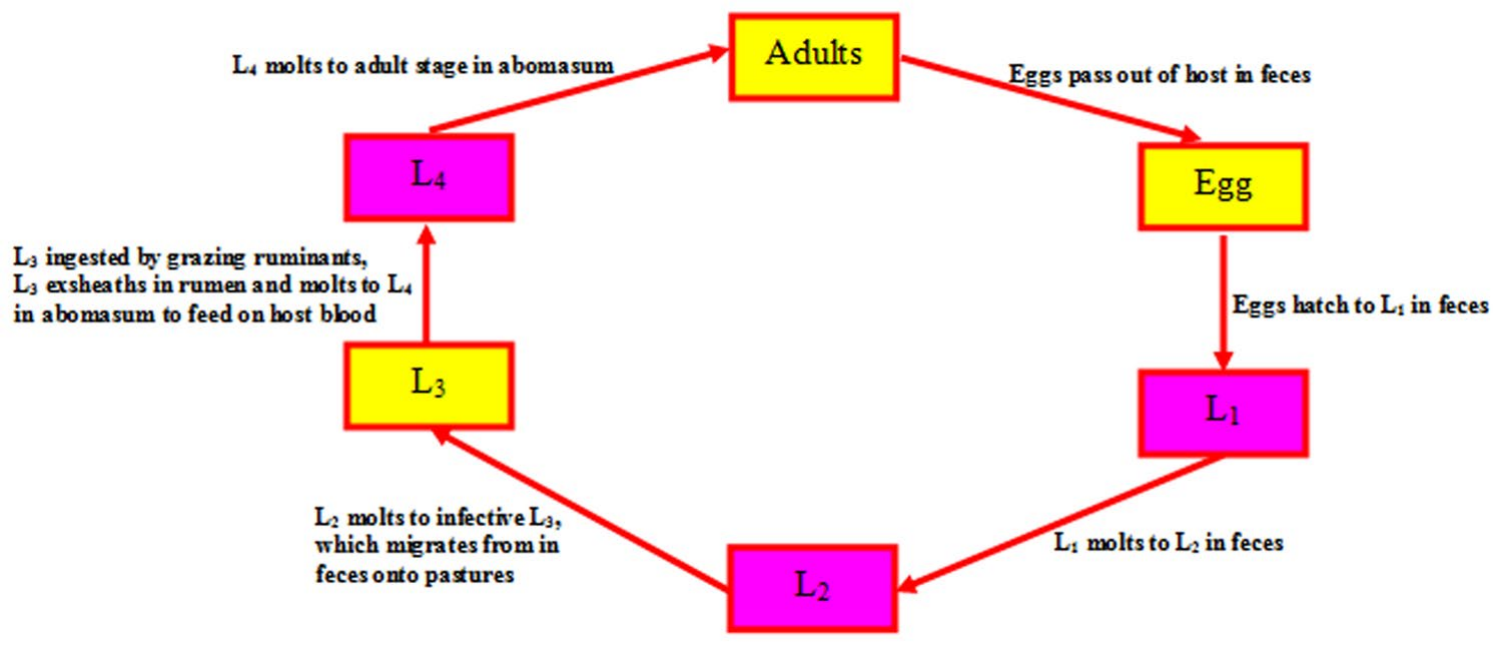
The basic life-cycle pattern of *H. contortus*.

**Figure 4:**
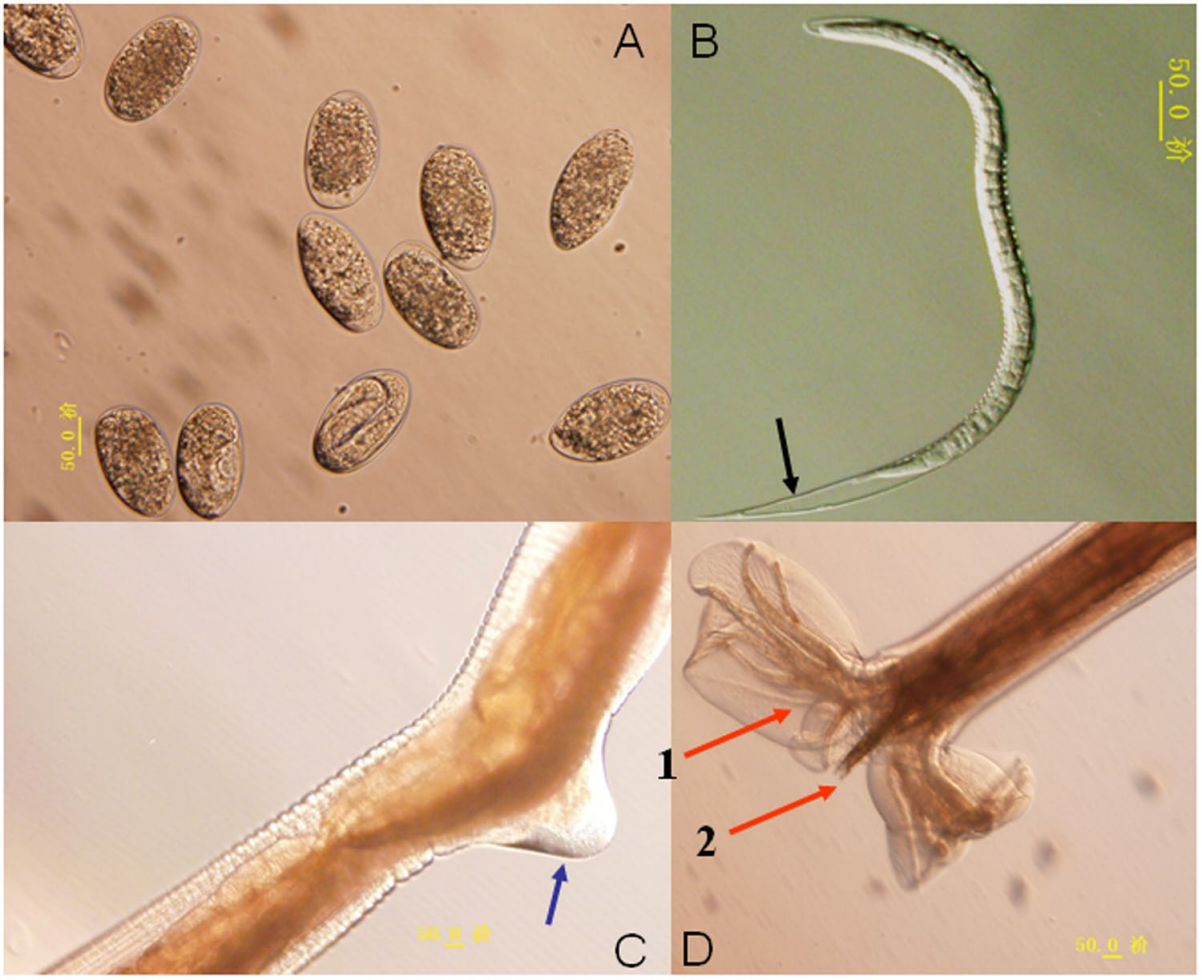
*H. contortus* life-cycle stages. (**A**) *H. contortus* egg. (**B**) *H. contortus* L_3_ larva. Slender larva, tail of sheath medium length (>50 µm <100 µm), tapering to point and often kinked. (**C**) *H. contortus* adult female vulval flap. The blue arrow is pointing to the vulval flap. (**D**) *H. contortus* adult male bursa. The red arrows are pointing to the capulatory bursa (1) spicules used to hold open the female worms genital opening (2).

The life-cycle of *H. contortus* consists of six stages: the egg, four larval stages (L_1_, L_2_, L_3_, L_4_) and the adult. Each stage in the life cycle is separated by a molt except for the egg - L_1_. The embryo develops to a 1^st^-stage larva within the egg, hatches and undergoes four molts before developing into the sexually mature adult. *H. contortus* passes through diverse environments during completion of its life-cycle. The adults and 4^th^-stage larvae are parasitic and adapted to the environment within the host, while the other stages are free-living (Fig. 3). Three distinct stages of the life-cycle of *H. contortus* are selected for the characterization of their microbiota, which are: eggs, L_3_ larval stage and adults (Fig. 4).

### Investigation of parasite surfaces for abomasal microbial contamination

Combining the evidence from the two distinct approaches, including SEM and next-generation sequencing of 16S rRNA gene (V3-V4 region), the obvious result is there is no contamination (i.e. intact bacteria and their DNA products) originating from the abomasal microbial community and consequently; the results of the *Haemonchus* microbiome are sufficient to remove an important obstacle about the abomasal bacterial contamination (Figure S1 and Table S1).

### Larval-stage of *H. contortus* displaying internalized transgenic *Escherichia coli*

**Figure 5:**
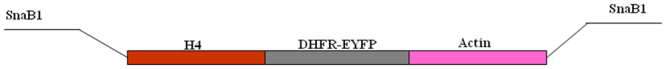
Schematic representation of YFP expressing plasmid: Expression cassette is shown as colored boxes.

**Figure 6:**
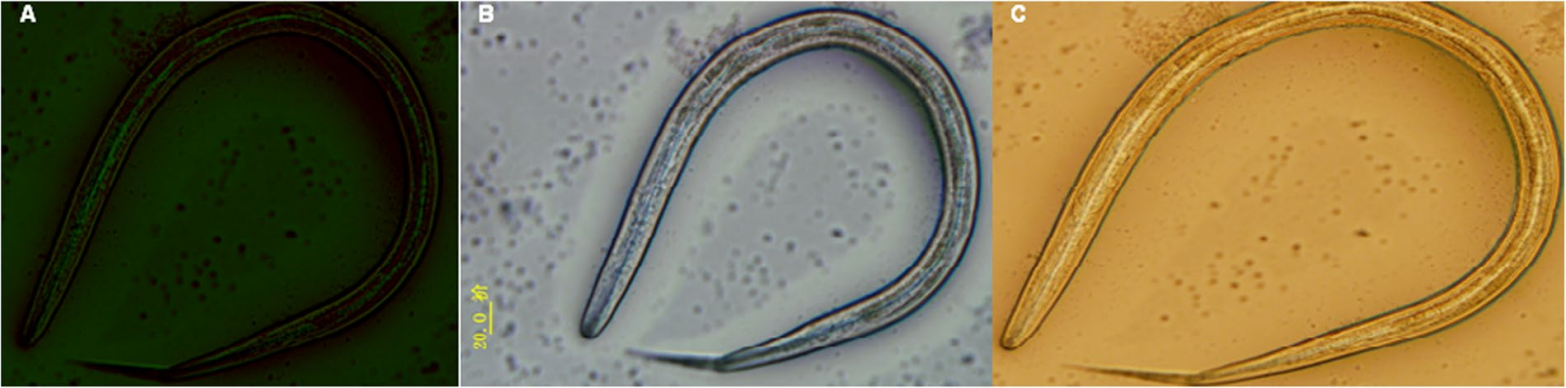
*H. contortus* showing internalized yellow fluorescent protein (YFP)-expressing *Escherichia coli.* Fluorescent (**A**), merged (**B**) and Bright (**C**) images were taken.

The expression cassette plasmid, H4-DHFR-EYFP-ACT (Fig. 5), was constructed from the recombinant plasmid pd2EYFP-N1+ formerly described by^21^. The mic1-EYFP and RFP were replaced with H4-DHFR. Therefore, the tandem dihydrofolate reductase thymidylate synthase- enhanced yellow fluorescent protein (DHFR-EYFP) coding region is flanked by the Histone 4 (H4) promoter from *E. tenella* and 3′region of actin. With respect to plasmid transformation, the procedure was conducted according to the manufacturer’s recommendations using BL21 (DE3) chemically competent cell (Beijing TransGen Biotech Co., Ltd.). A fluorescent microscopy image (Olympus, Tokyo, Japan) using the filter set at 488-nm excitation, 508-nm emission^22^ of the free-living larval-stage that incubated for 72 hours with yellow fluorescent protein (YFP)-expressing *E. coli* is presented in Fig. 6.

### Diversity index analysis of microbial community

Samples were evaluated for alpha diversity (microbial diversity within samples) and beta diversity (community diversity between samples) analysis.

### Alpha rarefaction curves

**Figure 7:**
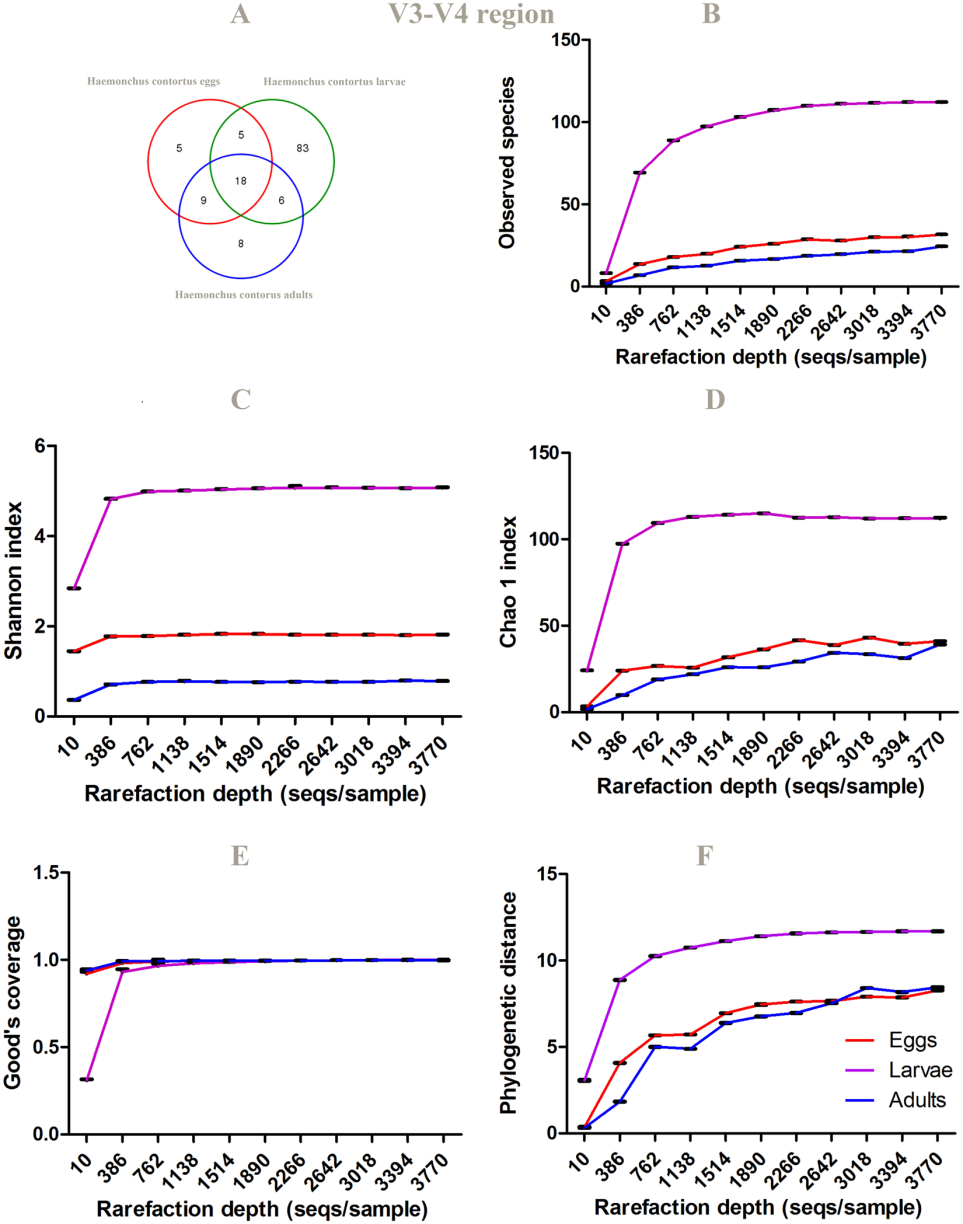
Venn graph **(A**) and alpha diversity analysis (**B–F**) of *H. contortus* eggs, larvae and adults based on 16S rRNA gene sequence (V3-V4 region) of parasite microbiome and 97% identity. Rarefraction curve for (**B**). Observed species (**C**). Shannon, (**D**). Chao1, (**E**). good’s coverage and (**F**). PD whole tree. Data represent average values from pooled populations of eggs/larvae/adults (male and female) come from three different sheep.

**Figure 8:**
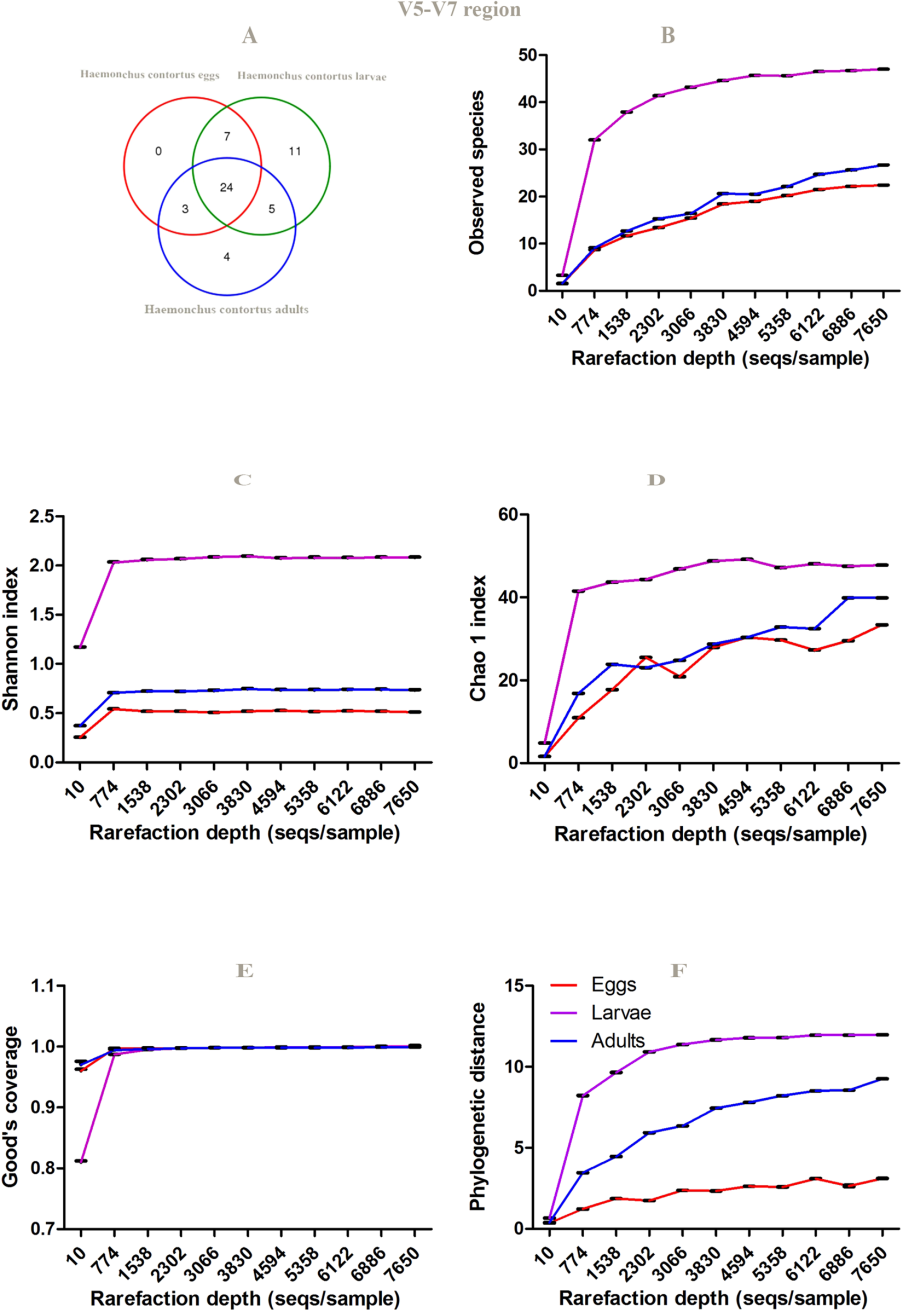
Venn graph (**A**) and alpha diversity analysis (**B–F**) of *H. contortus* eggs, larvae and adults based on 16S rRNA gene sequence (V5-V7 region) of parasite microbiome and 97% identity. Rarefraction curve for (**B**). observed species (**C**). Shannon, (**D**). Chao1, (**E**). good’s coverage and (**F**). PD whole tree. Data represent average values from pooled populations (i.e from each sheep) of eggs/larvae/adults (male and female) come from three different sheep.

It can be seen from the data in Table 2, Figs 7A–F and 8A–F, Tables S2 and S3 that V5–V6 amplicons showed reduced observed OTUs, regardless of *H. contortus* life- cycle stage, compared to V3-V4 amplicons, which could be due to greater conservation in this region of the 16S RNA gene sequence. The alpha diversity indexes represent species richness/number of OTUs per *H. contortus* life-cyle stages using various metrics. Alpha rarefaction curves of *H. contortus* life-cycle stages were computed exploiting the alpha_rarefaction.py in Qiime. Alpha diversity metrics, such as Phylogenetic diversity (PD) whole tree, Good’s coverage, Chao1, Shannon and Observed species were plotted using QIIME tools. They illustrated the number of observed species at a random pool of sequences in different depths (number of sequences). The rarefaction curves for all stages of the parasite reached the near plateau phase depicting satisfying sampling depth. The estimators, such as Shannon, Chao1, Good’s coverage and Observed species showed that the third larval-stage (L_3_) of *H. contortus* could significantly harbor a more abundant and diversified microbial community compared to those of the adult- and egg- stages, respectively. Furthermore, common microbial diversity indices, such as Simpson’s index, Shannon-Wiener species diversity index (H), richness (Table 2), were evaluated.

**Table 2:** Overview of alpha diversity metrics (interquartile range (IQR) and median) for the different life-cycle stage microbiome. *P*-values were calculated using Kruskal-Wallis test. The level of significance was determined at *P* < 0.05.

16S rRNA gene sequence region	Diversity index	*H. contortus* egg-stage microbiome^a^	*H. contortus* larval-stage Microbiome^a^	*H. contortus* adult -stage microbiome^a^	*H. contortus* eggs vs *H. contortus* larvae		*H. contortus* eggs vs *H. contortus* adults		*H. contortus* larvae vs *H. contortus* adults	
		Median, IQR	Median, IQR	Median, IQR	Median, IQR	Median, IQR	Chi-Square	Asymp. Sig.	Chi-Square	Asymp. Sig.
V3-V4 region	OTUs number of each sample (total species)	37.05, 0.45	111.8, 0.8	41.04, 0.64	3.8571	0.0495	3.8571	0.0495	3.8571	0.0495
	Shannon-Wiener species diversity index	1.22827, 0.000004	2.3507, 0	0.531, 0	4.5	0.0339	4.5	0.0339	5	0.0253
	Genus evenness (equitability)	0.340028, 0.00115	4.716712, 0.007137	0.143, 0.1302	3.8571	0.0495	0.4412	0.5066	3.9706	0.0463
	Simpson’s index	0.589393, 0.000393	0.825784, 0	0.771, 0	4.5	0.0339	4.5	0.0339	5	0.0253
V5-V7 region	OTUs number of each sample (total species)	34, 0.2	47.2, 0.6	36.1, 0.5	3.9706	0.0463	3.8571	0.0495	3.9706	0.0463
	Shannon-Wiener species diversity index	0.327, 0	1.25429, 0	0.48614, 0	5	0.0253	5	0.0253	5	0.0253
	Genus evenness (equitability)	0.0927, 0.0001	0.32542, 0.001084	0.013617, 0.121984	3.9706	0.0463	0.4412	0.5066	3.8571	0.0495
	Simpson’s index	0.121, 0	0.514703, 0	0.771735, 0.190294	5	0.0253	4.5	0.0339	4.5	0.0339

^a^Data represent median and interquartile range (IQR) values from pooled populations (i.e. from each sheep) of eggs/larvae/adults (male and female) come from three different sheep (natural infection).

Our results suggested that *H. contortus* larval-stage microbiome had been more taxon-rich compared to *H. contortus* egg and adult-stage microbiomes, respectively using two different amplification primer sets (targeting V3–V4, and V5–V7) and Illumina MiSeq. Moreover, *H. contortus* egg-stage microbiome compared to *H. contortus* larval-stage microbiome, and the later compared to *H. contortus* adult-stage microbiome displayed significant differences employing genus evenness, Simpson and Shannon-Wiener species diversity indices at the genus-level. Similarly, there was a consequential difference between *H. contortus* egg-stage microbiome in comparison with *H. contortus* adult-stage microbiome at the genus-level (targeting V3–V4, and V5–V7) regarding Simpson and Shannon-Wiener species diversity indices; however, genus evenness did not show any significant differences (Table 2).

### Beta diversity

**Figure 9:**
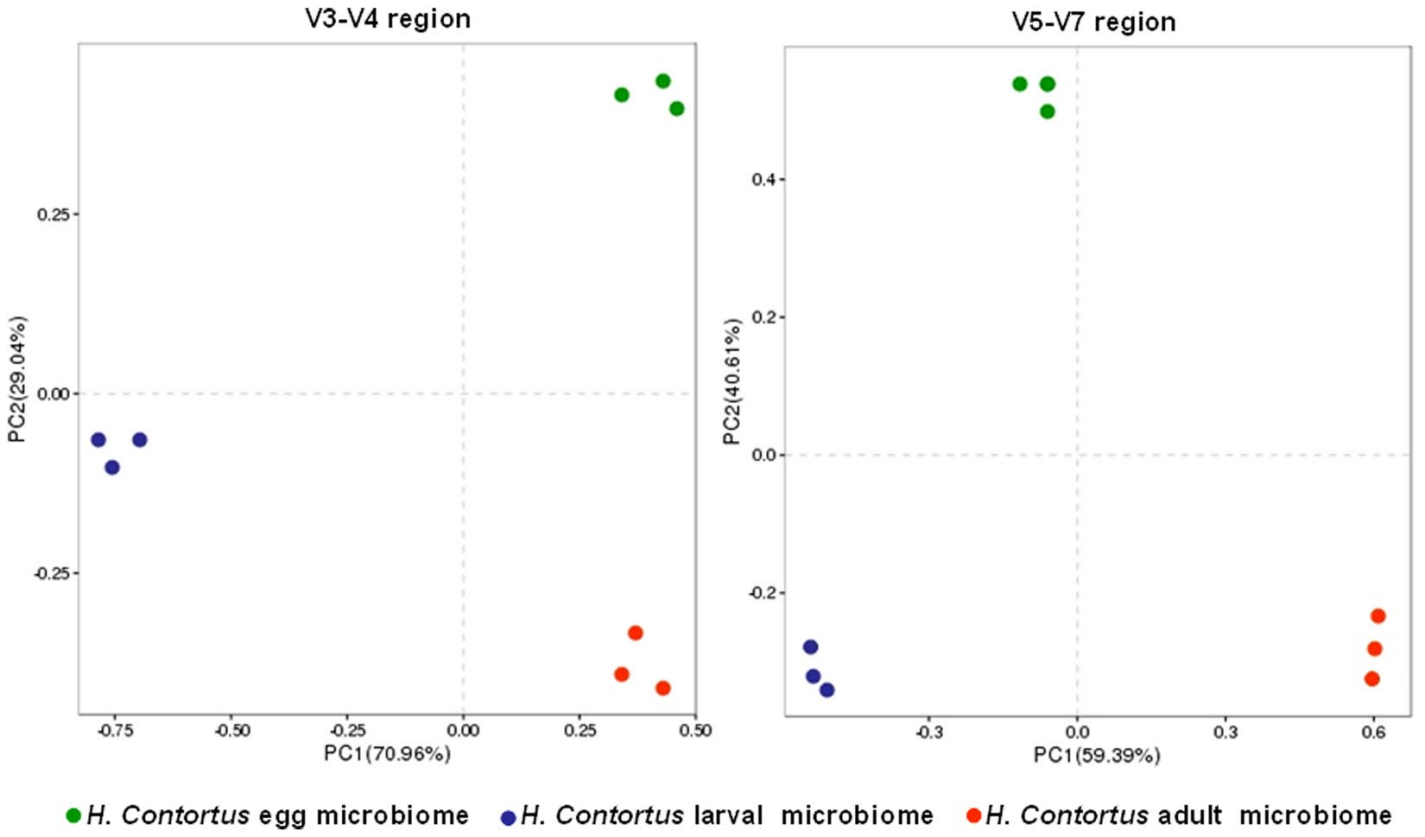
Principal coordinate analysis (PCoA) of *H. contortus* microbiome using primer pairs targeting V3–V4 (**A**) and V4–V5 (**B**) regions. The percentage variation explained with first two principal components (PC1 and PC2). Data represent average values from pooled populations (i.e. from each sheep) of eggs/larvae/adults (male and female) come from three different sheep.

Computing differences between microbial communities were calculated exploiting the default beta diversity metrics of weighted and unweighted UniFrac^23^ (beta_diversity_through_plots.py). Principal Coordinate Analysis (PCoA) was plotted using the result of UniFrac distance matrices to determine the similarity between groups of samples/time-points. The three-dimensional PCoA plots were visualized employing the Emperor tool^24^. The beta diversity analysis showed three very distinct clusters separating the different life-cycle stages of *H. Contortus* (Fig. 9).

#### The microbial community composition of the ovine abomasum and rumen in the context of *H. contortus* infection at the genus level

Two-month-old sheep (*Ovis aries*) that were raised under confined living conditions to avoid worm exposure were moved from a local farmer in Jin Zhan village, Chaoyang, Beijing, China with an average liveweight of 25.02 ± 2.2. Twelve parasite-naive sheep were divided into four treatments, uninfected-control groups and 7- and 50- day post-infection (dpi) *Haemonchus-* infected groups, in triplicate. 6 sheep were inoculated with 5,000 of *H. contortus* infective larvae laboratory strain and followed for 7 and 50 days. The animals were fed a fixed-formula diet containing forage and concentrate in a ratio of 1 to 1. The abomasal and ruminal microbial communities (V3-V4 amplicon) of the early (7-day post-infection) and late (50-day post-infection) *H. contortus* -infected sheep at the genus level are presented in Tables S4 and S5.

#### The analysis of microbial community structure and dominant taxa associated with distinct life-cycle stages of *H. contortus*

**Figure 10:**
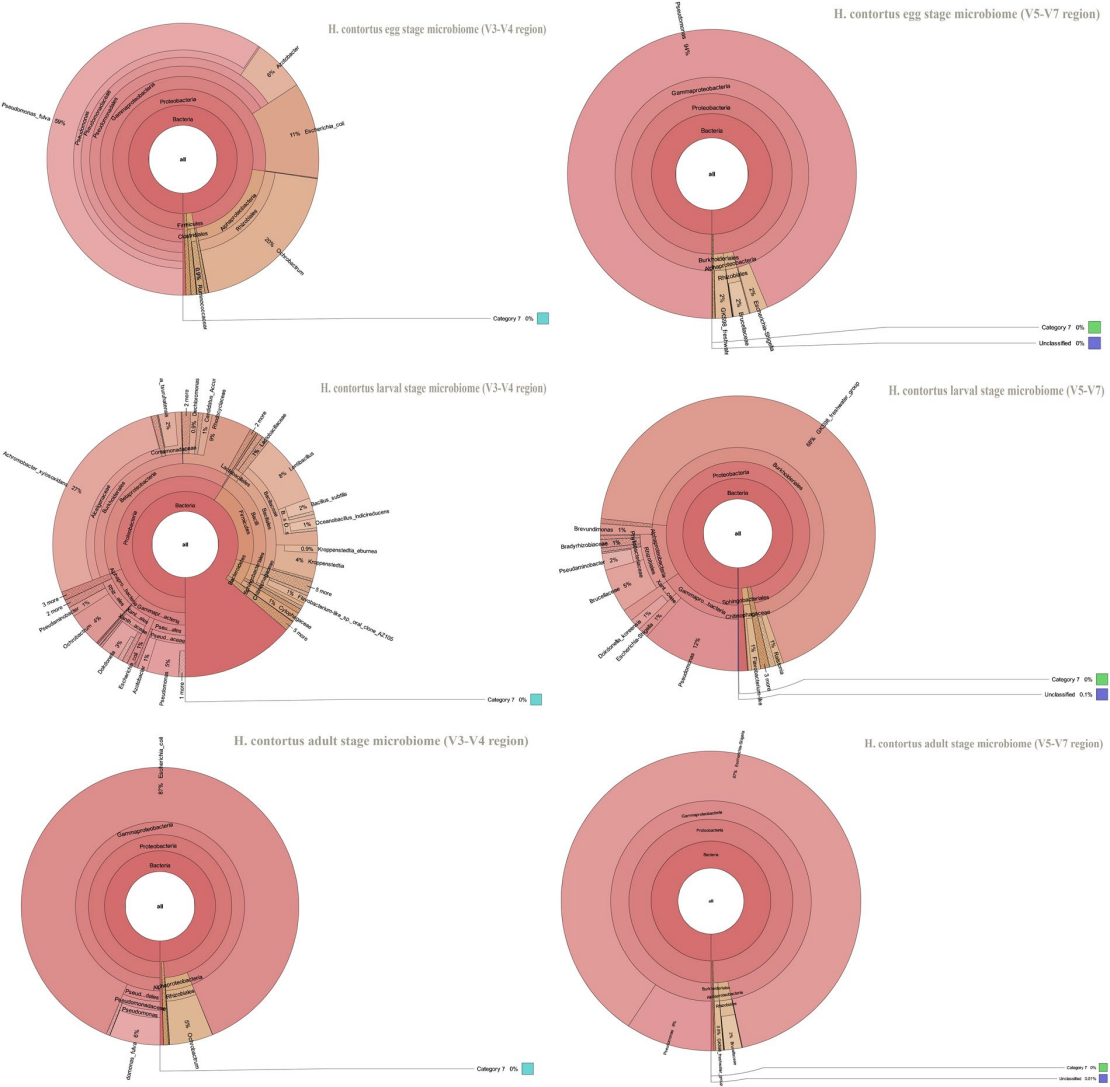
Krona plot of *H. contortus* egg-, larval-, adult- stage microbiome based on 16S rRNA gene sequence V3-V4 region (**A**) and V5-V7 region (**B**). Data represent average values from pooled populations (i.e. from each sheep) of eggs/larvae/adults (male and female) come from three different sheep.

To explore the bacterial community composition associated with the different life -cycle stages of *H. contortus*, we carried out high-throughput Illumina paired end sequencing. For a better survey, taxonomic abundances of all taxa can be visualized in a Krona plot^25^ that is based on the relative abundance of the microbial community associated with the different life-cycle stages of *H. contortus* (Fig. 10). Additionally, Kruskal-Wallis test of all microbial taxa are presented in Tables S6 and S7.

Four phyla for egg-stage microbiome (Proteobacteria 97.39%, Firmicutes 1.64%, Actinobacteria 0.323% and Bacteroidetes 0.31%) and four phyla for adult stage microbiome (Proteobacteria 99.02%, Firmicutes 0.37%, Tenericutes 0.131%, and Actinobacteria0. 0873%), eight for larval-stage microbiome (Proteobacteria 59.24%, Firmicutes 20.29%, Bacteroidetes 5.4%, Planctomycetes 0.29%, Acidobacteria 0.3972%, 0.2913%, Elusimicrobia 0.1059%, Candidate_division_WS3 0.1589%) using V3-V4 amplicons was identified. For all stages, the parasite microbiome was highly dominated (i.e. had a relative abundance greater than 1.0%) by microbes of the following bacterial phyla, Proteobacteria 97.39% and Firmicutes 1.64% for egg-stage microbiome, Proteobacteria 99.02% for adult-stage microbiome and Proteobacteria 59.24%, Firmicutes 20.29% and Bacteroidetes 5.4% for larval-stage microbiome. Targeting the hypervariable V5-V7 region, four phyla for egg-, larval- and adult- stage microbiome (Bacteroidetes, Proteobacteria, Gemmatimonadetes, Actinobacteria and Firmicutes) were characterized with different relative abundances. The predominant phyla observed in the three stages of *H. contortus* life-cycle were Proteobacteria for egg- and adult- stage microbiome with relative abundances 99.66% and 99.49% respectively, Proteobacteria 96.29% and Bacteroidetes 1.53% for larval-stage microbiome. Intriguingly, experimental sequencing of the V5-V7 region revealed phylum Gemmatimonadetes with relative abundances 0.46%, 0.019% and 0.015% for the larval-, egg- and adult- stage microbiome respectively, emphasizing the necessity for experimental sequencing of this region.

**Figure 11:**
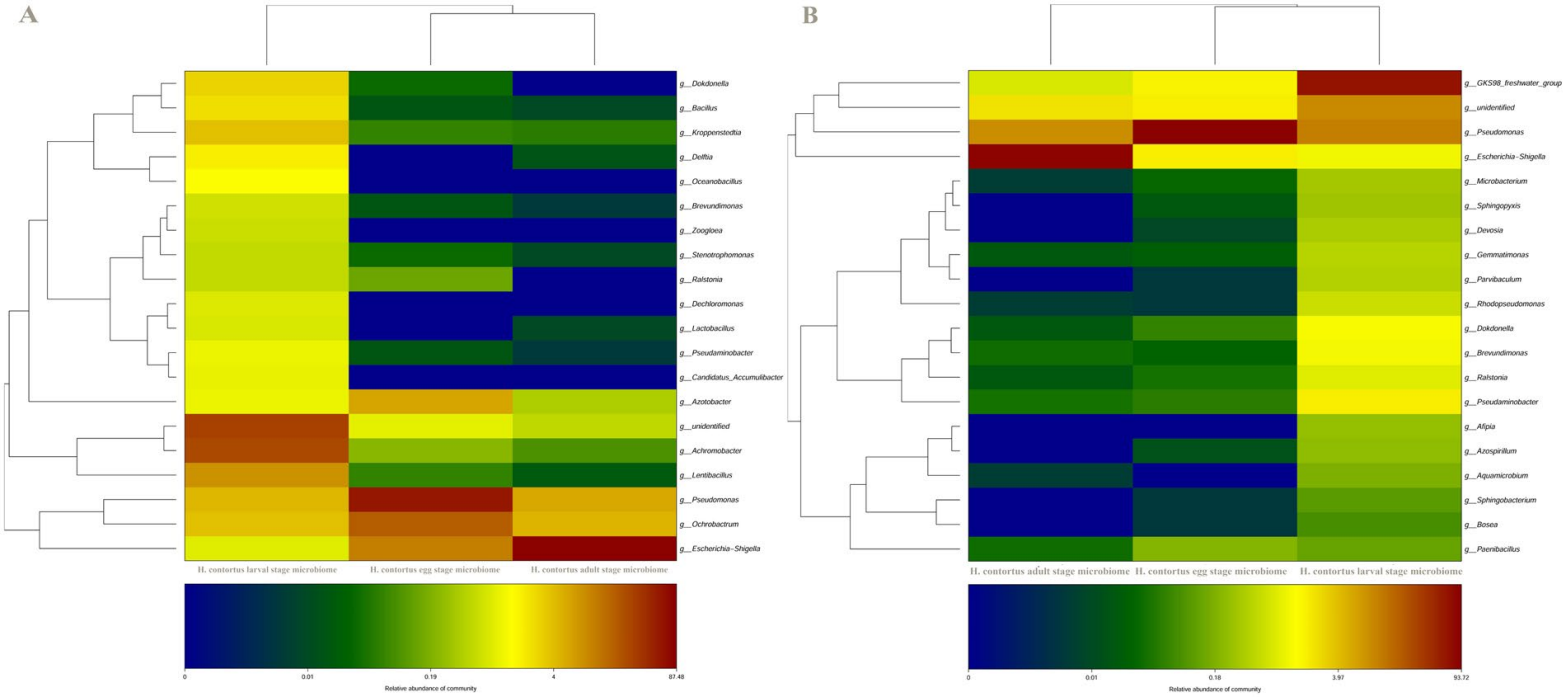
Double hierarchical dendrogram of the top 50 ranked *H. contortus* microbiota showing the relative abundance of the bacterial community at the genus level for different life-cycle stages of *H. contortus* using primer pairs targetingV3–V4 (**A**) and V4–V5 (**B**) regions. The relative values for bacterial species are depicted by color intensity, with legend indicated at the bottom of the figure. Data represent average values from pooled populations (i.e. from each sheep) of eggs/larvae/adults (male and female) come from three different sheep.

The results of the microbial community composition associated with the different life -cycle stages of ovine *H. contortus* (V3-V4/V5-V7 amplicons) at the genus level are set out in Tables S8 and S9. Furthermore, we assessed the relative distribution of bacterial taxa at the level of genus for each stage of *H. contortus* life-cycle using V3-V4 tags. *Pseudomonas* (59.58%), *Ochrobactrum* (19.64%), *Escherichia-Shigella* (11.44%) and *Azotobacter* (6.193%) were abundant in the egg-stage microbiome. The dominant genera of larval-stage microbiome are *Achromobacter* (26.67%), *Lentibacillus* (8.45%), *Pseudomonas* (4.69%), *Ochrobactrum* (4.212%), Kroppenstedtia (4.105%), Dokdonella (2.97), Bacillus (2.52%), *Delftia* (2.04%), *Oceanobacillus* (1.51%), Azotobacter (1.17%), *Pseudaminobacter* (1.17%) and *Candidatus_Accumulibacter* (1.09%). Additionally, the three most-abundant genera identified in the adult-stage microbiome are *Escherichia-Shigella* (87.5%), *Pseudomonas* (5.91%) and *Ochrobactrum* (5.06%). The representative members of genera, *Pseudomonas* and *Ochrobactrum* were prevalently shared in all the stages of the parasite life-cycle. Hierarchical clustering heatmap (Fig. 11A) depicts the relative percentage of each bacterial genus (variables clustering on the Y-axis) within each life -cycle stage (X-axis clustering). As can be seen from the heat map analysis below, the larval-stage microbiome using V3-V4 region did not cluster together indicating that the bacterial diversity of the larval-stage is varied and distinct is distinct from the other life- cycle stages. For V5-V7 tags, the dominant bacterial genera in the egg-stage belonged to *Pseudomonas* 93.7%, *Escherichia-Shigella* 2% and GKS98 freshwater group 1.84%, whereas in the adult-stage, the predominant genera belonged to GKS98 freshwater group 67.895%, *Pseudomonas* 11.5%, *Pseudaminobacter* 1.95%, *Dokdonella* 1.37%, *Brevundimonas* 1.28% and *Escherichia-Shigella* 1.2%. Furthermore, the adult-stage bacterial population was found to be prevalently composed of two genera: *Escherichia-Shigella* 87.32% and *Pseudomonas* 9.305%. Additionally, *Escherichia-Shigella* and *Pseudomonas* were *predominantly* shared by all stages. Hierarchically clustered heatmap analysis based on the bacterial community profiles at genus level disclosed that egg-stage microbiome and larval-stage microbiome clustered together (Fig. 11B).

#### The analysis of microbial community structure associated with individual adult female worms of *H. contortus*

The average individual-level data (single female adult worm) from the same population of sheep were consistent with the group-level data (clusters of male and female worms) collected during the winter 2017 and summer 2016 seasons, respectively with minor differences regarding the less common bacteria (Table S10).

## Discussion

The bacterial profiles of complex microbial communities associated with the different life-cycle stages of *H. contortus* field strains were explored with the aid of Illumina MiSeq sequencing technology. Furthermore, in this study the authors have advocated a set of measures that resulted in the absence of the cross-contamination by unwanted abomasal microbial community.

Bacteria are presumably transmitted vertically (from parents), horizontally (from the environment or unrelated animals) or in some cases a mixture of horizontally and vertically. As the barber’s pole worm, *H. contortus* is exposed to the microbiota of the rumen and abomasum; the nematode gut microbiota may reflect the external micro-organisms (i.e. from microhabitat [abomasum and rumen] and macrohabitat [pasture and manure]), which continually pass through its alimentary canal as evidenced by haemonchine larvae with internalized transgenic *Escherichia coli*. These results match those observed in earlier studies. Relatedly, significant changes in the abundance and composition of abomasal and ruminal microbiota were observed throughout *H. contortus* infections under the same diet regimen (forage (grass silage): concentrate (barley, maize, wheat, wheat bran and bruised soya) 1:1 ad libitum. The luminal abomasum microbiota is considerably dominated by microbes of the following bacterial phyla, Bacteroidetes 65.25%, Firmicutes 24.96%, Fibrobacteres 2.79%, Lentisphaerae 2.18%, Proteobacteria 1.41%, Spirochaetae 1.07% and Tenericutes 1.05% for control uninfected group, Bacteroidetes 71.23%, Firmicutes 18.43%, Proteobacteria 5.6% and Lentisphaerae 1.23% for 7-dpi group, Bacteroidetes 57.7%, Firmicutes 35.42%, Proteobacteria 2.86%, Fibrobacteres 1.14% for 50-dpi group. However, the highly dominant ruminal OTUs belonged to Bacteroidetes 79.82%, Firmicutes 14.33%, Proteobacteria 1.43% and Spirochaetae 1.3% for control uninfected group, Bacteroidetes 61.26%, Firmicutes 31.25%, Proteobacteria 2.23%, Lentisphaerae 1.36% and Spirochaetae 1.28% for 7-dpi group, Bacteroidetes 76.16%, Firmicutes 5.99%, Proteobacteria 15.75% and Spirochaetae 1.44% for 50- dpi group.

Similarly, for all stages, the parasite microbiome is highly dominated by microbes of the following bacterial phyla, Proteobacteria and Firmicutes for egg-stage microbiome, Proteobacteria for adult-stage microbiome and Proteobacteria, Firmicutes and Bacteroidetes for larval-stage microbiome using V3-V4 tags. However, the predominant phyla observed in the three stages of the *H. contortus* life-cycle were Proteobacteria for egg- and adult- stage microbiome and Proteobacteria 96.29% and Bacteroidetes 1.53% for larval stage microbiome employing V5-V7 tags. Furthermore, the free-living stages of *H. contortus*, such as L_1_ (rhabditiform), and L_2_ (rhabditiform) and L_3_ filiariform infective larvae on pasture may be a vector or reservoir, of environmental microbial community^26^. These results corroborate the ideas of^27^, who suggested that the representative members from these phyla are universal and are not limited to the peculiar environment. Among the aforementioned genera (see result section), *Pseudomonas*, *Ochrobactrum* and *Escherichia-Shigella* were predominantly shared by all the life-cycle stages of *H. contortus* (eggs, larvae and adults) using V3–V4 and V5–V7 amplicons.

*Ochrobactrum* spp., which are observed as emerging opportunistic pathogens, have been isolated from soil, hospital water sources, the rhizosphere of potato (*Solanum tuberosum*), rice (*Oryza sativa*), and wheat (*Triticum sativum*). The expression of AmpC beta-lactamases immensely complicates the therapy of infections caused by *Ochrobactrum* spp.^28^. Similarly, in 2009, Lacharme-Lora, *et al*.^26^ reported the potential role of helminths as pathogen vectors. Intriguingly, α-Proteobacteria group has the ability to avoid the host innate recognition^29,30^. Additionally, it has been demonstrated that *O. anthropi* infection provokes a mix of Th1 and Th2 type responses in the mice^30^. It can therefore be assumed that the *H. contortus* microbiome play a role in shaping the ovine immune response. Of special concern is the supporting evidence that the repression of Beta-2-microglobulin (beta-2-M), MHC class I alpha chain and MHC class II, DR alpha at day 7 post-infection and MHC class I alpha chain and MHC class II Alpha chain A alpha chain expression at day 50 post-infection in the abomasal tissue of sheep experimentally infected with *H. contortus* employing Illumina RNA-Seq technology (unpublished data).

There is a fierce competition for obtaining iron among microorganisms inside the ovine body to keep bacterial invaders under control. Iron is an indispensable element for many reduction–oxidation (redox) reactions and the virulence of pathogenic bacteria^31^. The predominant microbial genera, especially, *Pseudomonas* and *Escherichia* have developed an innovative way to confront with a variety of iron limitations by colonization of all the life-cycle stages of the blood-sucking parasite, *H. contortus* (eggs, larvae and adults). The probable association between the predominant microbial genera, such as *Pseudomonas* and *Escherichia,* and ovine hosts were further observed by data from *H. contortus* in naturally infected caprine hosts (unpublished data). The next few years will likely witness an immense number of studies uncovering the exact nature of this microbiome-*H. contortus* interaction, and finally reveal the specific molecular mechanisms underlying the nematode microbiome as an achilles’ heel of the gastrointestinal parasites. There is abundant room for further progress in comparing microbiome of different helminths, which has, and will continue to offer an important parallel goal for the removal of a wide-variety of devastating animal and human diseases.

## Electronic supplementary material

Supplementary materials
