# Erratum: Exploring the microbial community (microflora) associated with ovine *Haemonchus contortus* (macroflora) field strains

**DOI:** 10.1038/s41598-017-07623-9

**Published:** 2017-09-29

**Authors:** Saeed El-Ashram, Xun Suo

**Affiliations:** 1 State Key Laboratory for Agrobiotechnology & College of Veterinary Medicine, China Agricultural University, Beijing, 100193 China; 2 National Animal Protozoa Laboratory & College of Veterinary Medicine, China Agricultural University, Beijing, 100193 China; 3 Key Laboratory of Animal Epidemiology and Zoonosis of Ministry of Agriculture, Beijing, 100193 China; 4 Faculty of Science, Kafr El-Sheikh University, Kafr El-Sheikh, Egypt

*Scientific Reports* **7**:70; doi:10.1038/s41598-017-00171-2; Article published online 06 March 2017

This Article contains an error in Figure 5, where ‘T7 Promoter ’ is incorrectly given as ‘H4’ and 'T7 Terminator' is incorrectly given as 'Actin'. The correct Figure 5 appears below as Figure 1.

**Figure 1:**
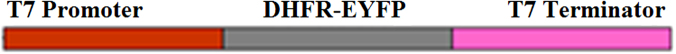
Schematic representation of YFP expressing plasmid: Expression cassette is shown as colored boxes.

There are in-text errors under the sub-heading “Larval-stage of *H. contortus* displaying internalized transgenic *Escherichia coli*”, where

“The expression cassette plasmid, H4-DHFR-EYFP-ACT (Fig. 5)”

should read:

“The expression cassette plasmid, T7 Promoter-DHFR-EYFP-T7 Terminator(Fig. 5)”

In addition,

“was constructed from the recombinant plasmid pd2EYFP-N1+ formerly described by^21^. The mic1-EYFP and RFP were replaced with H4-DHFR.”

should read:

“was constructed from the recombinant plasmid pd2EYFP-N1+ formerly described by^21^ and pET vector (Novagen, Madison,WI, USA).”

Additionally,

“coding region is flanked by the Histone 4 (H4) promoter from E. tenella and 3′region of actin”

should read:

“coding region is flanked by the T7 promoter of pET vector and Terminator of pET vector”

There is also an error in Table 2, where the column sub-headings for ‘*H. contortus* eggs vs *H. contortus* larva’ should read Chi-square and Asymp. Sig.

Furthermore, this Article contains an error in the legend of Figure 2:

“GoldViewTM Nucleic Acid Stained 1.5% Agarose gel of V3–V4 region of 16S RNA gene sequence (1, 2 and 3) and V5–V7 region of 16S RNA gene (4, 5 and 6) amplified product of eggs, L3larvae and adults of H. contortus. Lane 1 and 8: Direct-load™ Star Marker Plus (D2000 Plus); GenStar Biosolutions Co. Ltd, Beijing, China.”

should read:“GoldViewTM Nucleic Acid Stained 1.5% Agarose gel of V3–V4 region of 16S RNA gene sequence (1, 2 and 3) and V5–V7 region of 16S RNA gene (5, 6 and 7) amplified product of eggs, L3larvae and adults of H. contortus. Lane 1 and 8: Direct-load™ Star Marker Plus (D2000 Plus); GenStar Biosolutions Co. Ltd, Beijing, China.”

Finally, there is an error in the legend of Figure 11:

“Double hierarchical dendrogram of the top 50 ranked H. contortus microbiota showing the relative abundance of the bacterial community at the genus level for different life-cycle stages of H. contortus using primer pairs targetingV3–V4 (A) and V4–V5 (B) regions.”

should read:“Double hierarchical dendrogram of the top 20 ranked H. contortus microbiota showing the relative abundance of the bacterial community at the genus level for different life-cycle stages of H. contortus using primer pairs targetingV3–V4 (A) and V4–V5 (B) regions.”

