# Supplementary material for: Exploring the microbial community (microflora) associated with ovine Haemonchus contortus (macroflora) field strains
Source: Sci Rep. 2017 Mar 6;7:70. doi: 10.1038/s41598-017-00171-2 (PMC5427911; doi:10.1038/s41598-017-00171-2)
Supplement: Supplementary file 1 — Supplementary materials [file 41598_2017_171_MOESM1_ESM.pdf]

Supplementary Information

Exploring the microbial community (microflora) associated with ovine *Haemonchus contortus* (macroflora) field strains

Saeed El-Ashram [1,2,3,4,*], Xun Suo [1,2,3]

[1] State Key Laboratory for Agrobiotechnology & College of Veterinary Medicine, China Agricultural University, Beijing 100193, China

[2] National Animal Protozoa Laboratory & College of Veterinary Medicine, China Agricultural University, Beijing 100193, China

[3] Key Laboratory of Animal Epidemiology and Zoonosis of Ministry of Agriculture, Beijing 100193, China

[4] Faculty of Science, Kafr El-Sheikh University, Kafr El-Sheikh, Egypt

[*]Corresponding author, Email: saeed_elashram@yahoo.com(SE)

Supplementary material for "Exploring the microbial community (microflora) associated with ovine *Haemonchus contortus* (macroflora) field strains"

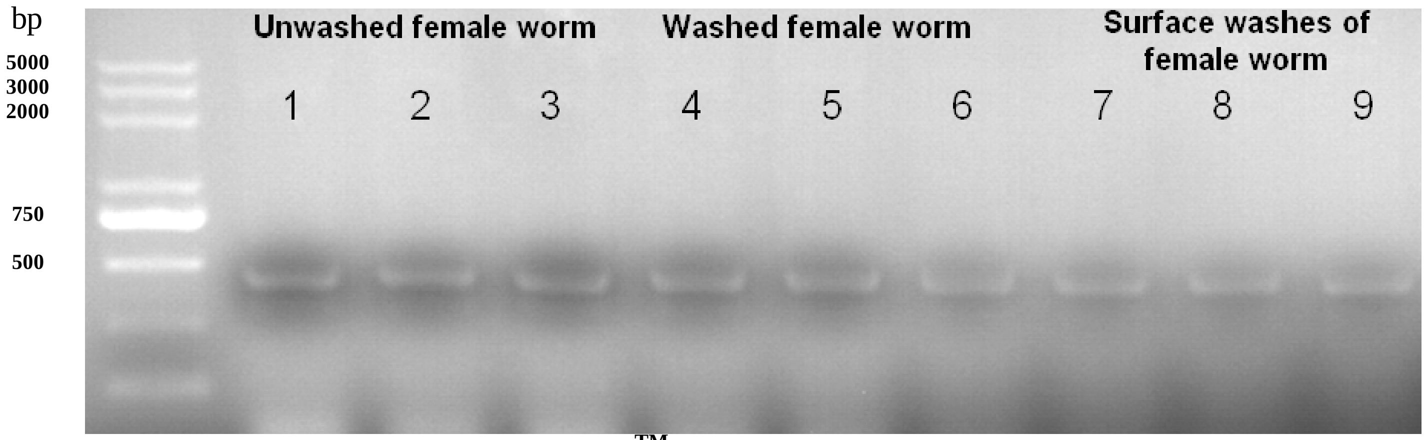

Supplementary Figure S1: GoldView™ Nucleic Acid Stained 1.5% Agarose gel of V3-V4 region of 16S rRNA gene amplified product of unwashed an individual female worm (1, 2 and 3), washed an individual female worm (5, 6 and 7) and surface washes of an individual female worm (7, 8 and 9). Lane 1: Direct-load™ Star Marker Plus (D2000 Plus); GenStar Biosolutions Co. Ltd, Beijing, China.

Supplementary Table S1: An average bacterial composition associated with *H. contortus* adult female worms using amplification of V3-V4 hypervariable region of bacterial 16S rRNA gene sequence.

	V3-V4 region		
	**Unwashed female worms[a]**	**Washed female Worms[a]**	**Washing solutions from female worms[a]**
	**(Average, ±SD)**	**(Average, ±SD)**	**(Average, ±SD)**
Anaerovibrio	**3.77E-03 ± 3.71E-04**	**0 ± 0**	**1.21E-03 ± 0.0003418**
Bacillus	**1.48E-03 ± 1.04E-04**	**0.00163131 ± 0.00015272**	**0.00E+00 ± 0**
Anaerotruncus	**2.57E-03 ± 2.56E-04**	**0 ± 0**	**8.24E-04 ± 0.000233**
Oribacterium	**5.21E-03 ± 5.17E-04**	**0 ± 0**	**1.67E-03 ± 0.0004729**
Ruminobacter	**2.36E-03 ± 2.36E-04**	**0 ± 0**	**7.57E-04 ± 0.0002131**
Mogibacterium	**2.36E-03 ± 2.36E-04**	**0 ± 0**	**7.57E-04 ± 0.0002131**
Acetitomaculum	**8.17E-03 ± 8.13E-04**	**0 ± 0**	**2.62E-03 ± 0.0007451**
Moryella	**1.45E-02 ± 1.46E-03**	**0 ± 0**	**4.64E-03 ± 0.001318**
Achromobacter	**2.52E-02 ± 1.77E-03**	**0.02773495 ± .002593656**	**0.00E+00 ± 0**
Kroppenstedtia	**7.41E-03 ± 5.20E-04**	**0.00815722 ± 0.00076303**	**0.00E+00 ± 0**
Stenotrophomonas	**4.44E-03 ± 3.12E-04**	**0.004894266 ± 0.0004577**	**0.00E+00 ± 0**
Butyrivibrio	**5.39E-02 ± 5.37E-03**	**0 ± 0**	**1.73E-02 ± 0.0049359**
Candidatus_Saccharimonas	**4.85E-03 ± 4.82E-04**	**0 ± 0**	**1.56E-03 ± 0.0004407**
Intestinimonas	**1.48E-03 ± 1.04E-04**	**0.00163131 ± 0.00015272**	**0.00E+00 ± 0**
Aeriscardovia	**2.22E-02 ± 1.56E-03**	**0.02447166 ± 0.00228877**	**0.00E+00 ± 0**
Saccharofermentans	**2.41E-02 ± 2.41E-03**	**0 ± 0**	**7.72E-03 ± 0.0021724**
Prevotella	**9.78E-01 ± 9.73E-02**	**0 ± 0**	**3.14E-01 ± 0.0888669**
Pseudomonas	**1.06E+00 ± 7.44E-02**	**1.16812319 ±**	**0.00E+00 ± 0**

		0.10924532	
Brevundimonas	1.48E-03 ± 1.04E-04	0.00163131 ± 0.00015272	0.00E+00 ± 0
Ochrobactrum	1.54E+00 ± 1.08E-01	1.699978513 ± 0.1589851	0.00E+00 ± 0
Rhizobium	1.48E-03 ± 1.04E-04	0.00163131 ± 0.00015272	0.00E+00 ± 0
Sphingomonas	1.48E-03 ± 1.04E-04	0.00163131 ± 0.00015272	0.00E+00 ± 0
Anaerosporobacter	3.83E-03 ± 3.82E-04	0 ± 0	1.23E-03 ± 0.0003514
Lentibacillus	4.44E-03 ± 3.12E-04	0.004894266 ± 0.000458	0.00E+00 ± 0
Atopobium	4.44E-03 ± 3.12E-04	0.004894266 ± 0.0004577	0.00E+00 ± 0
Luteibacter	2.96E-03 ± 2.08E-04	0.003262955 ± 0.000305	0.00E+00 ± 0
Ruminococcus	1.76E-01 ± 1.24E-02	0.19414332 ± 0.01815669	0.00E+00 ± 0
Selenomonas	6.35E-03 ± 6.27E-04	0 ± 0	2.04E-03 ± 0.0005729
Succiniclasticum	8.91E-02 ± 8.83E-03	0 ± 0	2.86E-02 ± 0.0080934
Succinivibrio	5.54E-02 ± 5.52E-03	0 ± 0	1.78E-02 ± 0 .0050292
Lactobacillus	1.48E-03 ± 1.04E-04	0.00163131 ± 0.00015272	0.00E+00 ± 0
Pyramidobacter	4.43E-03 ± 4.41E-04	0 ± 0	1.42E-03 ± 0.0004029
Delftia	2.96E-03 ± 2.08E-04	0.003262955 ± 0.000305	0.00E+00 ± 0
Acinetobacter	4.44E-03 ± 3.12E-04	0.004894266 ± 0.0004577	0.00E+00 ± 0
Bacteroides	2.81E-02 ± 2.76E-03	0 ± 0	9.00E-03 ± 0.0025395
Marvinbryantia	4.16E-03 ± 4.11E-04	0 ± 0	1.34E-03 ± 0.00038
Coprococcus	4.07E-03 ± 4.01E-04	0 ± 0	1.31E-03 ± 0.0003707
Mycoplasma	4.00E-02 ± 2.81E-03	0.044049058 ± 0.0041198	0.00E+00 ± 0
Arthrobacter	2.99E-05 ± 2.96E-06	0 ± 0	9.59E-06 ± 2.727E-06
Pseudaminobacter	1.48E-03 ± 1.04E-04	0.00163131 ± 0.00015272	0.00E+00 ± 0
Flavonifractor	4.44E-03 ± 3.12E-04	0.004894266 ± 0.0004577	0.00E+00 ± 0
Blautia	3.11E-03 ± 3.11E-04	0 ± 0	9.98E-04 ± 0.0002823
Staphylococcus	5.93E-03 ± 4.16E-04	0.00652591 ± 0.00061032	0.00E + 00 ± 0
Incertae_Sedis	4.07E-02 ± 4.06E-03	0 ± 0	1.31E-02 ± 0.0037072
Aquamicrobium	1.48E-03 ± 1.04E-04	0.00163131 ± 0.00015272	0.00E + 00±0
Syntrophococcus	4.67E-03 ± 4.62E-04	0 ± 0	1.50E-03 ± 0.0004236
Escherichia-Shigella	2.67E+01 ± 1.87E+00	29.4119116 ± 2.75065773	0.00E + 00±0
RC9_gut_group	2.96E-03 ± 2.08E-04	0.003262955 ± 0.000305	0.00E + 00±0
Desulfovibrio	4.49E-03 ± 4.47E-04	0 ± 0	1.44E-03 ± 0.0004122
Shinella	2.96E-03 ± 2.08E-04	0.003262955 ± 0.000305	0.00E+00 ± 0
Thalassospira	1.20E-03 ± 1.20E-04	0 ± 0	3.84E-04 ± 0.0001087
Shuttleworthia	1.08E-02 ± 1.07E-03	0 ± 0	3.45E-03 ± 0.0009794
Catonella	8.98E-04 ± 8.93E-05	0 ± 0	2.88E-04 ± 8.15E-05

[a]Data represent an average value from three female adult worm (each worm come from different sheep on one farm) ± standard deviation (SD).

Supplementary Table S2: Alpha diversity analysis of *H. contortus* microbiome based on 16S rRNA gene sequence (V3-V4) of *H. contortus* life-cycle stages.

	**Shannon Ave. ± SD[a]**	**Chao1 Ave. ± SD[a]**	**Good's coverage Ave. ± SD[a]**	**PD whole tree Ave. ± SD[a]**	**Observed species Ave. ± SD[a]**
**Eggs**	**1.447±0.003**	**3.35±0.05**	**0.92±0.002**	**0.36±0.003**	**3.3±0.01**
	**1.776±0.002**	**24±0.1**	**0.982±0.001**	**4.077±0.007**	**13.6±0.3**
	**1.782±0.002**	**26.743±0.4**	**0.99±0.002**	**5.674±0.004**	**17.9±0.1**
	**1.807±0.007**	**25.752±0.5**	**0.994±0.004**	**5.723±0.003**	**19.8±0.2**
	**1.829±0.007**	**31.796±0.1**	**0.994±0.003**	**6.954±0.004**	**24.1±0.1**
	**1.828±0.006**	**36.465±0.6**	**0.995±0.002**	**7.45±0.05**	**26±0.2**
	**1.813±0.003**	**41.645±0.4**	**0.995±0.004**	**7.622±0.01**	**28.6±0.3**
	**1.811±0.001**	**38.813±0.1**	**0.997±0.002**	**7.64±0.04**	**27.8±0.3**
	**1.813±0.003**	**43.134±0.3**	**0.997±0.004**	**7.906±0.006**	**29.9±0.3**
	**1.803±0.003**	**39.61±0.1**	**0.997±0.006**	**7.858±0.008**	**30±0.7**
	**1.815±0.005**	**41.055±0.14**	**0.997±0.005**	**8.274±0.004**	**31.5±0.4**
**Larvae**	**2.839±0.006**	**24.1±0.02**	**0.31±0.001**	**3.06±0.06**	**3.3±0.01**
	**4.824±0.004**	**97.442±0.4**	**0.93±0.03**	**8.878±0.008**	**13.6±0.3**
	**4.986±0.005**	**109.46±0.6**	**0.966±0.02**	**10.251±0.01**	**17.9±0.1**
	**5.006±0.006**	**113.016±0.25**	**0.98±0.01**	**10.747±0.01**	**19.8±0.2**
	**5.03±0.03**	**114.136±0.3**	**0.987±0.002**	**11.12±0.01**	**24.1±0.1**
	**5.059±0.008**	**115.011±0.17**	**0.992±0.002**	**11.406±0.006**	**26±0.2**
	**5.07±0.07**	**112.606±0.6**	**0.995±0.003**	**11.562±0.01**	**28.6±0.3**
	**5.07±0.02**	**112.757±0.2**	**0.997±0.002**	**11.625±0.01**	**27.8±0.3**
	**5.074±0.004**	**112.115±0.15**	**0.999±0.001**	**11.656±0.01**	**29.9±0.3**
	**5.065±0.001**	**112.186±0.18**	**0.999±0.001**	**11.683±0.01**	**30±0.7**
	**5.07±0.02**	**112±0.1**	**1±0.001**	**11.683±0.01**	**31.5±0.4**
**Adults**	**0.368±0.003**	**1.8±0.1**	**0.94±0.0001**	**0.315±0.004**	**1.8±0.01**
	**0.711±0.001**	**9.85±0.5**	**0.992±0.002**	**1.837±0.005**	**6.8±0.2**
	**0.766±0.003**	**18.975±0.5**	**0.992±0.001**	**5.001±0.008**	**11.5±0.4**
	**0.78±0.02**	**21.945±0.1**	**0.995±0.004**	**4.896±0.006**	**12.6±0.3**
	**0.765±0.004**	**26.013±0.1**	**0.995±0.003**	**6.387±0.007**	**15.7±0.2**
	**0.761±0.001**	**25.995±0.4**	**0.996±0.002**	**6.764±0.004**	**16.7±0.3**
	**0.772±0.002**	**29.268±0.1**	**0.996±0.001**	**6.964±0.003**	**18.5±0.4**

	**0.769±0.004**	**34.418±0.1**	**0.997±0.002**	**7.534±0.004**	**19.6±0.4**
	**0.767±0.005**	**33.54±0.4**	**0.997±0.001**	**8.405±0.005**	**21.2±0.2**
	**0.79±0.009**	**31.303±0.1**	**0.997±0.002**	**8.176±0.006**	**21.3±0.2**
	**0.788±0.003**	**39.218±0.1**	**0.997±0.0005**	**8.44±0.04**	**24.3±0.3**

[a]Data represent average values from pooled populations (i.e from each sheep) of eggs/larvae/adults (male and female) come from three different sheep ± standard deviation (SD).

Supplementary Table S3: Alpha diversity analysis of *H. contortus* microbiome based on 16S rRNA gene sequence (V5-V7) of *H. contortus* life-cycle stages.

	Shannon Ave. ± SD[a]	Chao1 Average. ± SD[a]	Good's coverage Ave. ± SD[a]	PD whole tree Ave. ± SD[a]	Observed species Ave. ± SD[a]
**Eggs**	**0.255±0.004**	**1.6±0.03**	**0.96±0.005**	**0.36±0.01**	**1.5±0.04**
	**0.543±0.003**	**10.95±0.01**	**0.997±0.0003**	**1.225±0.005**	**8.7±0.06**
	**0.519±0.003**	**17.783±0.007**	**0.997±0.0004**	**1.866±0.006**	**11.7±0.055**
	**0.52±0.005**	**25.483±0.008**	**0.997±0.0003**	**1.749±0.006**	**13.4±0.04**
	**0.507±0.004**	**20.868±0.009**	**0.998±0.0004**	**2.381±0.009**	**15.4±0.065**
	**0.52±0.004**	**27.928±0.009**	**0.998±0.0003**	**2.342±0.008**	**18.4±0.06**
	**0.528±0.0015**	**30.31±0.01**	**0.998±0.00035**	**2.631±0.009**	**19±0.07**
	**0.517±0.002**	**29.69±0.01**	**0.998±0.00036**	**2.584±0.007**	**20.2±0.02**
	**0.523±0.003**	**27.249±0.009**	**0.999±0.0005**	**3.097±0.01**	**21.5±0.04**
	**0.519±0.004**	**29.524±0.008**	**0.999±0.0004**	**2.65±0.08**	**22.2±0.01**
	**0.512±0.002**	**33.372±0.008**	**0.999±0.0002**	**3.108±0.008**	**22.4±0.03**
**Larvae**	**1.168±0.006**	**4.85±0.01**	**0.81±0.004**	**0.663±0.003**	**3.3±0.03**
	**2.031±0.009**	**41.501±0.001**	**0.987±0.0007**	**8.222±0.01**	**32±0.05**
	**2.058±0.009**	**43.685±0.012**	**0.995±0.0006**	**9.648±0.011**	**37.9±0.06**
	**2.067±0.005**	**44.309±0.004**	**0.997±0.0007**	**10.924±0.011**	**41.4±0.02**
	**2.086±0.006**	**46.844±0.014**	**0.998±0.0009**	**11.38±0.01**	**43.2±0.05**
	**2.094±0.004**	**48.76±0.01**	**0.998±0.0008**	**11.662±0.012**	**44.6±0.04**
	**2.073±0.013**	**49.176±0.009**	**0.999±0.0006**	**11.789±0.01**	**45.7±0.04**
	**2.081±0.009**	**47.145±0.011**	**0.999±0.0008**	**11.799±0.01**	**45.6±0.05**
	**2.078±0.008**	**48.098±0.011**	**0.999±0.0009**	**11.955±0.011**	**46.5±0.03**
	**2.082±0.008**	**47.537±0.012**	**1±0.001**	**11.953±0.009**	**46.7±0.05**
	**2.083±0.007**	**47.75±0.01**	**1±0.003**	**11.976±0.009**	**47±0.06**
**Adults**	**0.373±0.001**	**1.6±0.02**	**0.97±0.01**	**0.391±0.001**	**1.6±0.01**
	**0.709±0.002**	**16.883±0.01**	**0.994±0.004**	**3.464±0.003**	**9.1±0.07**
	**0.724±0.003**	**23.842±0.012**	**0.996±0.003**	**4.462±0.002**	**12.7±0.02**
	**0.723±0.003**	**23.01±0.01**	**0.997±0.002**	**5.929±0.005**	**15.3±0.04**
	**0.731±0.01**	**24.768±0.011**	**0.998±0.001**	**6.355±0.005**	**16.4±0.08**
	**0.746±0.013**	**28.73±0.01**	**0.998±0.001**	**7.444±0.004**	**20.6±0.07**
	**0.737±0.01**	**30.32±0.01**	**0.998±0.001**	**7.804±0.004**	**20.5±0.06**
	**0.737±0.011**	**32.829±0.011**	**0.998±0.001**	**8.216±0.006**	**22.1±0.08**
	**0.74±0.01**	**32.424±0.011**	**0.998±0.001**	**8.516±0.006**	**24.7±0.065**
	**0.741±0.011**	**39.875±0.012**	**0.999±0.0005**	**8.555±0.005**	**25.6±0.057**
	**0.736±0.007**	**39.86±0.01**	**0.999±0.0006**	**9.256±0.006**	**26.7±0.056**

[a]Data represent average values from pooled populations (i.e from each sheep) of eggs/larvae/adults (male and female) come from three different sheep ± standard deviation (SD) (i.e. each cluster represents a sheep) ± standard deviation (SD)

Supplementary Table S4: Ovine abomasal microbiome composition at the genus level in the context of *Haemonchus* infection.

**Genus**	**Control group[a]**	**7-day post-infection group[a]**	**50-day post-infection group[a]**
Achromobacter	**0**	**0.4862853±0.00103244**	**0**
Solobacterium	**0.2671057±0.000298942**	**0.6483818±0.001376589**	**0.3896459±0.000268505**
Desulfobulbus	**0**	**0.4862853±0.00103244**	**0.08646436±0.0000595825**
Delftia	**0**	**0.2161007±0.000458807**	**0**
Clostridium_sensu_stricto_1	**0**	**0.1080504±0.000229403**	**0**
Chryseobacterium	**0**	**8.266853±0.01755148**	**0**
Lysinimonas	**0**	**0.1080504±0.000229403**	**0**
Anaerotruncus	**1.468376±0.001643391**	**0.4322517±0.00091772**	**3.723269±0.0025657**
Kineococcus	**0**	**0.1080504±0.000229403**	**0**
Roseburia	**9.611181±0.01075673**	**5.673331±0.01204513**	**0.04323218±0.0000297912**
Flavonifractor	**70.748970±0.07918149**	**49.006770±0.104047**	**197.722900±0.1362506**
Oribacterium	**2.358299±0.002639383**	**3.187871±0.006768217**	**7.533127±0.005191069**
Ruminobacter	**6.095981±0.006822556**	**2.701586±0.005735777**	**3.420212±0.002356864**
Weissella	**0**	**3.187871±0.006768217**	**0.04323218±0.0000297912**
possible_genus_Sk003-Sk004	**0**	**0.1080504±0.000229403**	**0**
Mogibacterium	**6.985904±0.007818549**	**2.107238±0.004473908**	**5.931254±0.00408722**
Acetitomaculum	**2.936749±0.003286778**	**2.647552±0.005621058**	**11.819220±0.008144608**
Moryella	**2.002330±0.002240986**	**9.671676±0.02053408**	**20.910920±0.01440969**
Mucilaginibacter	**0**	**0.1080504±0.000229403**	**0**
Modestobacter	**0**	**0.1080504±0.000229403**	**0**
Myroides	**0.0444619±0.0000497613**	**0.3241511±0.00068821**	**0**
Syntrophomonas	**0.1333857±0.000149284**	**0**	**0.08646436±0.0000595825**
Sanguibacter	**0**	**0.3241511±0.00068821**	**0**
Bosea	**0**	**0.3781763±0.000802912**	**0**
Howardella	**0.4449633±0.000497998**	**0.1080504±0.000229403**	**0.5628193±0.000387838**
Clostridiales_bacterium_canine_oral_taxon_162	**0.1333857±0.000149284**	**0.05402518±0.000114702**	**0**
Butyrivibrio	**35.285490±0.03949115**	**151.775100±0.322236**	**77.928890±0.0537007**
Comamonas	**0.0444619±0.0000497613**	**19.451420±0.0412976**	**0**
Methylobacterium	**0**	**0.4862853±0.00103244**	**0**
Pseudoramibacter	**0**	**0.1620755±0.000344105**	**0**
Patulibacter	**0**	**0.7024111±0.0014913**	**0**
Brevibacillus	**0.0444619±0.0000497613**	**0.05402518±0.000114702**	**0**
Janthinobacterium	**0**	**0.7564447±0.001606019**	**0**
Campylobacter	**0.0889238±0.0000995226**	**0.3241511±0.00068821**	**0.04323218±0.0000297912**
Solibacillus	**0**	**0**	**0.2597282±0.000178978**
Wohlfahrtiimonas	**0.1778476±0.000199045**	**0**	**0.1300317±0.0000896047**
Stenotrophomonas	**0**	**6.807998±0.01445416**	**0.08646436±0.0000595825**
U29-B03	**2.091321±0.002340584**	**0.810474±0.00172073**	**11.646040±0.008025272**
Olsenella	**0**	**0.3781763±0.000802912**	**0.5628193±0.000387838**
Rhizobium	**0**	**5.295109±0.01124213**	**0**
Paenibacillus	**0.3559693±0.000398397**	**0.5943482±0.00126187**	**0.3463501±0.000238669**
Psychrobacter	**0**	**0.1080504±0.000229403**	**0**
Actinobaculum	**0.0444619±0.0000497613**	**0.2161007±0.000458807**	**0.04323218±0.0000297912**
Paraprevotella	**0.2671057±0.000298942**	**1.242730±0.002638459**	**0.2597282±0.000178978**

Gluconobacter	**0**	**0.2701259±0.000573508**	**0**
Bacillus	**0.4894587±0.000547797**	**1.945141±0.004129758**	**0.6494077±0.000447506**
Fibrobacter	**93.264050±0.1043801**	**18.208690±0.03865914**	**38.271740±0.02637301**
Candidatus_Captivus	**0.0444619±0.0000497613**	**0.05402518±0.000114702**	**0**
Candidatus_Saccharimonas	**11.391030±0.01274872**	**11.076500±0.02351669**	**7.013600±0.004833064**
Empedobacter	**0**	**9.887806±0.02099295**	**0**
Saccharofermentans	**21.224690±0.02375445**	**21.504620±0.04565679**	**34.894820±0.02404598**
Serratia	**0**	**0.7024111±0.0014913**	**0**
Kandleria	**0**	**27.880370±0.05919323**	**0**
Bordetella	**0**	**0.1080504±0.000229403**	**0**
Duganella	**0**	**0.2161007±0.000458807**	**0**
Citrobacter	**0.0444619±0.0000497613**	**14.210340±0.0301702**	**0**
Oscillibacter	**2.135819±0.002390386**	**5.511234±0.01170099**	**1.342108±0.000924845**
Megasphaera	**0**	**2.809649±0.005965207**	**0**
RC9_gut_group	**216.429600±0.2422256**	**93.691000±0.1989168**	**112.260900±0.07735885**
Victivallis	**11.702500±0.01309732**	**14.480500±0.03074377**	**0.5628193±0.000387838**
Sphingobium	**0**	**0.4862853±0.00103244**	**0**
dgA-11_gut_group	**0.0889238±0.0000995226**	**0**	**0**
Bifidobacterium	**0.6674433±0.000746995**	**0.1620755±0.000344105**	**0.04323218±0.0000297912**
Enterorhabdus	**4.983574±0.005577563**	**3.025774±0.006424067**	**4.156208±0.002864038**
Lactococcus	**0.0444619±0.0000497613**	**7.240250±0.01537188**	**0**
Hafnia	**0**	**0.3241511±0.00068821**	**0**
Ethanoligenens	**0.2671057±0.000298942**	**0**	**0**
Enterobacter	**0**	**9.023298±0.0191575**	**0**
Prevotella	**1288.966000±1.442597**	**2320.122000±4.925886**	**1415.318000±0.9752943**
Selenomonas	**1.201397±0.001344591**	**2.431426±0.005162198**	**9.178292±0.00632475**
Intestinimonas	**2.224810±0.002489983**	**1.404826±0.002982608**	**4.978789±0.003430877**
Succiniclasticum	**43.294810±0.04845509**	**39.010900±0.08282463**	**128.885700±0.088815**
Sharpea	**0**	**47.493880±0.100835**	**0.3029604±0.00020877**
Succinivibrio	**15.707160±0.01757929**	**27.069890±0.05747249**	**80.223460±0.05528189**
Arcobacter	**0**	**0.3781763±0.000802912**	**0**
SP3-e08	**0.9789173±0.001095594**	**0**	**0.08646436±0.0000595825**
Iamia	**0**	**0.1620755±0.000344105**	**0**
Solirubrobacter	**0**	**0.2161007±0.000458807**	**0**
Ochrobactrum	**0.0444619±0.0000497613**	**0.6483818±0.001376589**	**0.04323218±0.0000297912**
Pyramidobacter	**0.4894587±0.000547797**	**6.753964±0.01433944**	**6.407485±0.00441539**
Bacteroides	**15.707160±0.01757929**	**3.133842±0.006653506**	**40.652900±0.02801387**
Jatrophihabitans	**0**	**0.1080504±0.000229403**	**0**
Deltaproteobacteria_bacterium_canine_oral_taxon_266	**0**	**0.1080504±0.000229403**	**0**
Arthrobacter	**0**	**0.9185412±0.001950169**	**0.04323218±0.0000297912**
Treponema	**34.573550±0.03869435**	**33.661760±0.07146779**	**29.786150±0.0205256**
Alloprevotella	**1.201397±0.001344591**	**3.728190±0.007915376**	**0.3896459±0.000268505**
Microvirga	**0**	**0.1080504±0.000229403**	**0**
Sphingomonas	**0**	**7.240250±0.01537188**	**0**
Anaerovorax	**7.697843±0.008615343**	**3.512060±0.007456507**	**4.719027±0.003251876**
Elusimicrobium	**6.896913±0.007718951**	**11.184570±0.02374612**	**2.943981±0.002028694**
Mitsuokella	**0.1778476±0.000199045**	**0.2161007±0.000458807**	**2.814100±0.001939193**
Staphylococcus	**0.1333857±0.000149284**	**0**	**0.00000000000000**

Schwartzia	**0.1333857±0.000149284**	**0**	**3.117154±0.002148027**
Mesorhizobium	**0**	**0.3781763±0.000802912**	**0**
Nocardioides	**0**	**0.3241511±0.00068821**	**0**
Aeromonas	**0**	**0.9185412±0.001950169**	**0**
Xanthomonas	**0**	**0.7564447±0.001606019**	**0**
Pseudobutyrivibrio	**15.484680±0.01733029**	**12.157140±0.025811**	**4.632439±0.003192208**
Christensenella	**0.8009326±0.000896395**	**0.2161007±0.000458807**	**0.4762309±0.00032817**
Actinotalea	**0**	**0.1080504±0.000229403**	**0**
Methanobrevibacter	**0.2226438±0.000249181**	**0**	**0**
Escherichia-Shigella	**0.00000000000000**	**0.1080504±0.000229403**	**0**
Marvinbryantia	**2.135819±0.002390386**	**0.2701259±0.000573508**	**6.017842±0.004146888**
Anaerofustis	**0.3115676±0.000348703**		**0.1300317±0.0000896047**
probable_genus_10	**7.831332±0.008764743**	**3.620127±0.007685946**	**0.9091694±0.000626508**
Coprococcus	**1.957835±0.002191188**	**0.9185412±0.001950169**	**5.887961±0.004057387**
Deinococcus	**0**	**0.1080504±0.000229403**	**0**
Pseudomonas	**0.2226438±0.000249181**	**13.129710±0.02787588**	**0.08646436±0.0000595825**
Papillibacter	**10.812580±0.01210132**	**0.1620755±0.000344105**	**0.3896459±0.000268505**
Cellulomonas	**0**	**0.1080504±0.000229403**	**0**
Lapillicoccus	**0**	**0.1620755±0.000344105**	**0**
Defluviimonas	**0**	**0.1620755±0.000344105**	**0**
Streptococcus	**1.201397±0.001344591**	**0.1620755±0.000344105**	**0.1732638±0.000119396**
Roseomonas	**0**	**0.1080504±0.000229403**	**0**
Ruminococcus	**107.324800±0.1201168**	**67.269490±0.1428209**	**96.069000±0.06620103**
Denitrobacterium	**0.1333857±0.000149284**	**0**	**0**
Succinimonas	**0.3115676±0.000348703**	**0**	**0.04323218±0.0000297912**
Candidatus_Hepatincola	**0.2671057±0.000298942**	**0**	**0.1732638±0.000119396**
Nitrobacter	**0**	**0.3781763±0.000802912**	**0**
Variovorax	**0**	**0.4322517±0.00091772**	**0**
Atopobium	**0.8009326±0.000896395**	**5.295109±0.01124213**	**11.386280±0.00784627**
Phocaeicola	**0.3115676±0.000348703**	**3.187871±0.006768217**	**3.420212±0.002356864**
Fretibacterium	**0.3115676±0.000348703**	**2.269334±0.004818057**	**0.3029604±0.00020877**
Quinella	**0**	**0**	**0.2597282±0.000178978**
Enhydrobacter	**0**	**1.674982±0.003556179**	**0**
Stomatobaculum	**0.2226438±0.000249181**	**0.3781763±0.000802912**	**0**
Kocuria	**0**	**0.1620755±0.000344105**	**0**
Anaeroplasma	**24.917880±0.02788782**	**4.484634±0.009521395**	**0.08646436±0.0000595825**
Leuconostoc	**0**	**8.861202±0.01881335**	**0**
Kurthia	**0.0444619±0.0000497613**	**0.1080504±0.000229403**	**0**
Soonwooa	**0**	**0.2701259±0.000573508**	**0**
Massilia	**0**	**0.1620755±0.000344105**	**0**
Oscillospira	**0**	**0.3781763±0.000802912**	**0.5195268±0.000358005**
Brevundimonas	**0**	**1.242730±0.002638459**	**0**
Pedobacter	**0**	**0.3781763±0.000802912**	**0**
Sporobacter	**0.3115676±0.000348703**	**0.1080504±0.000229403**	**0.2597282±0.000178978**
Thalassospira	**5.829002±0.006523757**	**11.346660±0.02409027**	**1.731754±0.001193349**
Acinetobacter	**0.2671057±0.000298942**	**54.193820±0.1150597**	**1.125639±0.000775676**
SR1_bacterium_canine_oral_taxon _380	**0**	**0.6483818±0.001376589**	**0**
Alysiella	**0**	**0**	**0.08646436±0.0000595825**

Lactobacillus	**0**	**7.726535±0.01640432**	**0.04323218±0.0000297912**
Shuttleworthia	**1.067908±0.001195191**	**0.1620755±0.000344105**	**15.585780±0.01074014**
Possible_genus_Sk018	**1.201397±0.001344591**	**1.026604±0.002179599**	**0**
Pantoea	**0**	**0.5943482±0.00126187**	**0**
Devosia	**0**	**0.6483818±0.001376589**	**0**
Curtobacterium	**0**	**2.701586±0.005735777**	**0**
Alkaliphilus	**0.0444619±0.0000497613**	**0.1080504±0.000229403**	**0.04323218±0.0000297912**
Collinsella	**0**	**0**	**0.08646436±0.0000595825**
Catonella	**0.1333857±0.000149284**	**0.2161007±0.000458807**	**1.298815±0.000895012**
Enterococcus	**0.1333857±0.000149284**	**1.945141±0.004129758**	**0.1732638±0.000119396**
Sphingobacterium	**0.0444619±0.0000497613**	**4.916886±0.01043911**	**0**
Aeromicrobium	**0**	**1.134667±0.002409029**	**0**
Anaerosporobacter	**0.1333857±0.000149284**	**0**	**5.541608±0.003818716**
Lachnospira	**0**	**0.1080504±0.000229403**	**0**
Desulfovibrio	**0.4004647±0.000448196**	**4.484634±0.009521395**	**6.494073±0.004475058**
Aeriscardovia	**0.1333857±0.000149284**	**0.6483818±0.001376589**	**0.6927001±0.000477339**
Wautersiella	**0**	**14.642600±0.03108792**	**0**
Hydrogenoanaerobacterium	**0.0444619±0.0000497613**	**0**	**0.1732638±0.000119396**
Spirochaeta	**1.112407±0.001244994**	**0.5943482±0.00126187**	**0.04323218±0.0000297912**
Vagococcus	**0**	**0.1620755±0.000344105**	**0**
Syntrophococcus	**3.515201±0.003934176**	**3.674156±0.007800657**	**6.753839±0.004654062**
Incertae_Sedis	**29.100520±0.03256899**	**30.473890±0.06469957**	**58.836310±0.04054403**
Acetobacter	**0**	**1.188696±0.002523739**	**0**
Anaerovibrio	**0.2671057±0.000298942**	**1.242730±0.002638459**	**5.455023±0.00375905**
Blautia	**7.475362±0.008366346**	**8.428950±0.01789563**	**4.502558±0.003102707**

[a]Each value is an average of three samples collected from three experimentally *H. contortus*-infected sheep (i.e. each sample represents a sheep) ± standard deviation (SD)

Supplementary Table S5: Ovine ruminal microbiome composition at the genus level in the context of *Haemonchus* infection.

**Genus**	**Control group[a]**	**7-day post-infection group[a]**	**50-day post-infection group[a]**
Leuconostoc	**0**	**5.350628±0.03108216**	**0**
Denitrobacterium	**0.4538265±0.003355203**	**0**	**0**
Wohlfahrtiimonas	**0.2475418±0.001830111**	**0**	**0**
Bacteroides	**15.182560±0.1122468**	**0.5827417±0.003385186**	**1.778635±0.01738169**
Megasphaera	**0**	**0.3178591±0.001846465**	**0**
Lachnospira	**0**	**0.4238122±0.002461953**	**0.03176134±0.000310387**
Pseudobutyrivibrio	**40.514330±0.2995281**	**17.164390±0.09970912**	**5.113575±0.04997235**
Pyramidobacter	**0**	**0.9535774±0.005539395**	**0.285852±0.002793485**
Spirochaeta	**0.1650278±0.001220074**	**0.4238122±0.002461953**	**0.06352267±0.000620775**
Atopobium	**0.7838822±0.005795351**	**0.6357182±0.00369293**	**0.190568±0.001862324**
Elusimicrobium	**3.176786±0.02348642**	**0.105953±0.000615488**	**0.1270453±0.001241549**
Brevundimonas	**0**	**0.3708356±0.002154209**	**0**
Succinivibrio	**41.504500±0.3068486**	**5.138722±0.02985119**	**408.450800±3.991580**
Solobacterium	**0**	**0.4767887±0.002769698**	**0**
Sphingobium	**0**	**0.2648826±0.001538721**	**0**
Victivallis	**3.671869±0.02714664**	**16.104860±0.09355423**	**0.06352267±0.000620775**
Stenotrophomonas	**0**	**1.854178±0.01077105**	**0**
U29-B03	**1.155195±0.008540517**	**0.3708356±0.002154209**	**4.097213±0.04003996**
Pseudomonas	**0.2062848±0.001525092**	**1.748225±0.01015556**	**0**
Treponema	**43.484830±0.3214895**	**28.236480±0.1640276**	**38.304170±0.374327**
Wautersiella	**0.04125696±0.000305018**	**1.695249±0.009847814**	**0**
Paracoccus	**0**	**0.105953±0.000615488**	**0**
Candidatus_Saccharimonas	**19.885850±0.1470189**	**10.330420±0.06001012**	**1.111647±0.01086355**
Chryseobacterium	**0**	**0.5297652±0.003077442**	**0**
Papillibacter	**1.155195±0.008540517**	**1.695249±0.009847814**	**0**
Ruminobacter	**0**	**0**	**6.225222±0.0608359**
Acinetobacter	**0.2887987±0.002135129**	**12.555440±0.07293537**	**0**
Ochrobactrum	**0**	**0.1589296±0.000923233**	**0**
Oribacterium	**2.640445±0.01952118**	**7.204807±0.04185321**	**3.684315±0.03600492**
Klebsiella	**0**	**1.801202±0.0104633**	**0**
Microvirga	**0**	**0.105953±0.000615488**	**0**
Arthrobacter	**0**	**0.105953±0.000615488**	**0**
Enterorhabdus	**4.208210±0.03111188**	**11.813760±0.06862696**	**0.7305108±0.007138907**
RC9_gut_group	**188.503100±1.393629**	**56.049160±0.3255934**	**15.404250±0.1505378**
Succiniclasticum	**60.317670±0.445937**	**34.752600±0.2018802**	**10.195390±0.09963431**
probable_genus_10	**5.280891±0.03904236**	**2.330967±0.01354074**	**0.09528401±0.000931162**
Photorhabdus	**0**	**0**	**0.1270453±0.001241549**
Marvinbryantia	**0.04125696±0.000305018**	**0.05297652±0.000307744**	**0**
Syntrophococcus	**0.990167±0.007320443**	**4.026216±0.02338856**	**0.2223294±0.002172711**
Sphingomonas	**0**	**2.119061±0.01230977**	**0**

Thalassospira	**0.6188544±0.004575277**	**1.059530±0.006154884**	**0.190568±0.001862324**
Empedobacter	**0**	**1.112507±0.006462628**	**0**
Desulfovibrio	**0.8663961±0.006405388**	**3.549427±0.02061886**	**1.619828±0.01582975**
Paenibacillus	**0.1650278±0.001220074**	**0.105953±0.000615488**	**0**
Incertae_Sedis	**12.995940±0.09608082**	**21.296560±0.1237132**	**3.684315±0.03600492**
Oscillibacter	**0.04125696±0.000305018**	**0.3178591±0.001846465**	**0.06352267±0.000620775**
Streptococcus	**2.021591±0.0149459**	**0.4238122±0.002461953**	**0**
Selenomonas	**10.561780±0.07808473**	**8.582196±0.04985456**	**24.487990±0.2393086**
Gluconobacter	**0**	**0.105953±0.000615488**	**0**
Bacillus	**0.4538265±0.003355203**	**0.2648826±0.001538721**	**0.03176134±0.000310387**
Enterococcus	**0.08251392±0.000610037**	**0.8476243±0.004923907**	
Succinimonas	**0**	**0**	**0.09528401±0.000931162**
Methylobacterium	**0**	**0.2119061±0.001230977**	**0**
Devosia	**0**	**1.112507±0.006462628**	**0**
Variovorax	**0**	**0.105953±0.000615488**	**0**
Pseudoxanthomonas	**0.08251392±0.000610037**	**0.105953±0.000615488**	**0**
Sphingobacterium	**0**	**0.9006009±0.005231651**	**0**
Blvii28_wastewater-sludge_group	**0.04125696±0.000305018**	**0**	**0.03176134±0.000310387**
possible_genus_Sk018	**0.6601114±0.004880295**	**2.489897±0.01446398**	**0.03176134±0.000310387**
Alloprevotella	**2.145362±0.01586096**	**0**	**0.1588067±0.001551936**
Schwartzia	**0.9489101±0.007015425**	**0**	**1.111647±0.01086355**
Enhydrobacter	**0**	**0.3708356±0.002154209**	**0**
Campylobacter	**0.2475418±0.001830111**	**0.05297652±0.000307744**	**0.06352267±0.000620775**
Anaerobiospirillum	**0.1237709±0.000915055**	**0**	**0.2223294±0.002172711**
Roseburia	**9.489100±0.07015425**	**3.072638±0.01784916**	**0**
Faecalibacterium	**0**	**0**	**0.1588067±0.001551936**
Intestinimonas	**0.2887987±0.002135129**	**6.516112±0.03785253**	**0.2223294±0.002172711**
Anaeroplasma	**2.062848±0.01525092**	**4.079192±0.0236963**	**0.03176134±0.000310387**
Comamonas	**0.08251392±0.000610037**	**2.225014±0.01292526**	**0**
Rhizobium	**0**	**0.4238122±0.002461953**	**0**
Anaerotruncus	**0.2062848±0.001525092**	**0.2119061±0.001230977**	**0**
Methanobrevibacter	**0.7013683±0.005185314**	**0**	**0.03176134±0.000310387**
Anaerovibrio	**3.094272±0.02287639**	**3.337521±0.01938788**	**7.559198±0.07387216**
Mogibacterium	**3.960668±0.02928177**	**1.589296±0.009232325**	**0.3811361±0.003724647**
Hafnia	**0**	**0.105953±0.000615488**	**0**
Weissella	**0**	**3.496450±0.02031112**	**0**
Acetitomaculum	**0.6188544±0.004575277**	**6.357183±0.0369293**	**0.285852±0.002793485**
Rhodopseudomonas	**0**	**0.105953±0.000615488**	**0**
Kandleria	**0**	**0.2648826±0.001538721**	**0**
Desulfobulbus	**0.08251392±0.000610037**	**0.3178591±0.001846465**	**0.1588067±0.001551936**
Butyrivibrio	**31.644090±0.2339492**	**101.609000±0.5902534**	**10.640050±0.1039797**
Saccharofermentans	**8.994017±0.06649403**	**21.296560±0.1237132**	**2.159771±0.02110633**
hoa5-07d05_gut_group	**0.08251392±0.000610037**	**0**	**0**
Enterobacter	**0**	**6.833971±0.039699**	**0**

Blautia	**2.929244±0.02165631**	**17.111420±0.09940137**	**0.6669881±0.006518132**
Bosea	**0**	**0.105953±0.000615488**	**0**
Paraprevotella	**0.04125696±0.000305018**	**0.9006009±0.005231651**	**0.1270453±0.001241549**
Fretibacterium	**0.7838822±0.005795351**	**2.330967±0.01354074**	**0.2540907±0.002483098**
Olsenella	**0**	**0.1589296±0.000923233**	**0**
Ruminococcus	**60.400190±0.446547**	**74.537960±0.4329961**	**22.201180±0.2169607**
Curtobacterium	**0**	**0.3178591±0.001846465**	**0**
Lactobacillus	**0**	**7.204807±0.04185321**	**0**
Alkaliphilus	**0**	**0.05297652±0.000307744**	**0.03176134±0.000310387**
Fibrobacter	**31.520320±0.2330341**	**5.297652±0.03077442**	**11.211750±0.1095667**
Mesorhizobium	**0**	**0.105953±0.000615488**	**0**
Anaerovorax	**0.7013683±0.005185314**	**1.907155±0.01107879**	**0.4128974±0.004035034**
Flavonifractor	**3.176786±0.02348642**	**5.456582±0.03169765**	**4.573633±0.04469576**
Lactococcus	**0**	**6.675042±0.03877577**	**0**
Prevotella	**2024.685000±14.968780**	**1098.574000±6.381691**	**1833.995000±17.922690**
Aeromicrobium	**0**	**0.4238122±0.002461953**	**0**
Moryella	**0**	**0.1589296±0.000923233**	**0.1270453±0.001241549**
Subdoligranulum	**0**	**0**	**0.06352267±0.000620775**
Howardella	**2.516675±0.01860613**	**3.496450±0.02031112**	**0.4764201±0.004655809**
Coprococcus	**0.04125696±0.000305018**	**0.2119061±0.001230977**	**0.03176134±0.000310387**
Shuttleworthia	**0.4538265±0.003355203**	**0.3708356±0.002154209**	**2.255055±0.02203749**
Veillonella	**0**	**0**	**0.06352267±0.000620775**
Candidatus_Hepatincola	**0.2475418±0.001830111**	**0**	**0.03176134±0.000310387**
SR1_bacterium_canine_oral_taxon_380	**0**	**0.2648826±0.001538721**	**0**
Quinella	**0**	**0**	**0.3493747±0.00341426**

[a]Each value is an average of three samples collected from three experimentally *H. contortus*-infected sheep (i.e. each sample represents a sheep) ± standard deviation (SD)

Supplementary Table S6: An average composition of significantly different microbial taxa associated with *H. contortus* eggs, larvae and adults using 16S rRNA gene (V3-V4 region). *P*-values were calculated using Kruskal-Wallis test. The level of significance was determined at P < 0.05.

**Phylum**									
	**Eggs**	**Larvae**	**Adults**	**Eggs vs adults**		**Eggs vs larvae**		**Larvae vs adults**	
**V3-V4 region**	**Median, IQR[a]**	**Median, IQR[a]**	**Median, IQR[a]**	**Chi-square**	***p*-value**	**Chi-square**	***p*-value**	**Chi-square**	***p*-value**
**Planctomycetes**	**0**	**0.3256886, 0.00233**	**0**	**4.3548**	**0.0369**	**0**	**0**	**4.3548**	**0.0369**
**Elusimicrobia**	**0**	**0.1184322, 0.000847**	**0**	**4.3548**	**0.0369**	**0**	**0**	**4.3548**	**0.0369**
**Candidate_**	**0**	**0.1776483, 0.0012712**	**0**	**4.3548**	**0.0369**	**0**	**0**	**4.3548**	**0.0369**
**Firmicutes**	**0.607866, 0.00738**	**22.679767, 0.162288**	**0.153335, 0.0023912**	**3.8571**	**0.0495**	**3.8571**	**0.0495**	**3.8571**	**0.0495**
**Bacteroidetes**	**0.11323, 0.001375**	**6.0400424, 0.04322**	**0**	**3.8571**	**0.0495**	**4.3548**	**0.0369**	**4.3548**	**0.0369**
**Tenericutes**	**0**	**0**	**0.0537668, 0.0008384**	**0**	**0**	**4.3548**	**0.0369**	**4.3548**	**0.0369**
**Acidobacteria**	**0**	**0.4441208, 0.003178**	**0**	**4.3548**	**0.0369**	**0**	**0**	**4.3548**	**0.0369**
**Spirochaetae**	**0**	**0.2960805, 0.0021186**	**0**	**4.3548**	**0.0369**	**0**	**0**	**4.3548**	**0.0369**
**Proteobacteria**	**36.08457, 0.43828**	**66.23321, 0.47394**	**40.639736, 0.633758**	**3.8571**	**0.0495**	**3.8571**	**0.0495**	**3.8571**	**0.0495**
**Actinobacteria**	**0.119189, 0.001448**	**0**	**0.0358445, 0.000559**	**4.3548**	**0.0369**	**3.8571**	**0.0495**	**4.3548**	**0.0369**

**Class**									
	**Eggs**	**Larvae**	**Adults**	**Eggs vs adults**		**Eggs vs larvae**		**Larvae vs adults**	
**V3-V4 region**	**Median, IQR[a]**	**Median, IQR[a]**	**Median, IQR[a]**	**Chi-square**	***p*-value**	**Chi-square**	***p*-value**	**Chi-square**	***p*-value**
**OM190**	**0**	**0.32568856, 0.00233**	**0**	**4.3548**	**0.0369**	**0**	**0**	**4.3548**	**0.0369**
**Acidobacteria**	**0**	**0.44412076, 0.00317796**	**0**	**4.3548**	**0.0369**	**0**	**0**	**4.3548**	**0.0369**
**Bacilli**	**0.238379, 0.002896**	**22.6797669, 0.1622882**	**0.047793, 0.0007453**	**3.8571**	**0.0495**	**3.8571**	**0.0495**	**3.8571**	**0.0495**
**Elusimicrobia**	**0**	**0.1184322, 0.00084746**	**0**	**4.3548**	**0.0369**	**0**	**0**	**4.3548**	**0.0369**
**Sphingobacteriia**	**0**	**4.02669492, 0.02881356**	**0**	**4.3548**	**0.0369**	**0**	**0**	**4.3548**	**0.0369**
**Deltaproteobacteria**	**0**	**0.29608051, 0.0021186**	**0**	**4.3548**	**0.0369**	**0**	**0**	**4.3548**	**0.0369**
**Coriobacteriia**	**0.047676, 0.000579**	**0**	**0.005974, 0.000093164**	**4.3548**	**0.0369**	**3.8571**	**0.0495**	**4.3548**	**0.0369**
**Spirochaetes**	**0**	**0.29608051, 0.0021186**	**0**	**4.3548**	**0.0369**	**0**	**0**	**4.3548**	**0.0369**
**Gammaproteobacteria**	**28.64119, 0.34787**	**13.0571504, 0.0934322**	**38.50898, 0.60052987**	**3.8571**	**0.0495**	**3.8571**	**0.0495**	**3.8571**	**0.0495**
**Clostridia**	**0.369487, 0.004488**	**0**	**0.105542, 0.00164588**	**4.3548**	**0.0369**	**3.8571**	**0.0495**	**4.3548**	**0.0369**
**Flavobacteriia**	**0**	**0.65137712, 0.004661**	**0**	**4.3548**	**0.0369**	**0**	**0**	**4.3548**	**0.0369**
**Actinobacteria**	**0.071514, 0.000868**	**0**	**0.02987, 0.000465816**	**4.3548**	**0.0369**	**3.8571**	**0.0495**	**4.3548**	**0.0369**
**Betaproteobacteria**	**0.148987, 0.00181**	**44.5601165, 0.318856**	**0.04381, 0.0006832**	**3.8571**	**0.0495**	**3.8571**	**0.0495**	**3.8571**	**0.0495**
**Cytophagia**	**0**	**1.30275424, 0.009322**	**0**	**4.3548**	**0.0369**	**0**	**0**	**4.3548**	**0.0369**
**Bacteroidia**	**0.11323, 0.001375**	**0**	**0**	**4.3548**	**0.0369**	**4.3548**	**0.0369**	**0**	**0**
**Alphaproteobacteria**	**7.294386, 0.088595**	**8.31986229, 0.0595339**	**2.086948, 0.032545**	**3.8571**	**0.0495**	**3.8571**	**0.0495**	**3.8571**	**0.0495**
**Mollicutes**	**0**	**0**	**0.053767, 0.000838469**	**0**	**0**	**4.3548**	**0.0369**	**4.3548**	**0.0369**

Order									
	Eggs	Larvae	Adults	Eggs vs adults		Eggs vs larvae		Larvae vs adults	
V3-V4	Median, IQR[a]	Median, IQR[a]	Median, IQR[a]	Chi-square	*p*-value	Chi-square	*p*-value	Chi-square	*p*-value
Rhodocyclales	0	9.6522246, 0.069068	0	4.3548	0.0369	0	0	4.3548	0.0369
Clostridiales	0.369487, 0.004488	0	0.105542, 0.001646	4.3548	0.0369	3.8571	0.0495	4.3548	0.0369
Myxococcales	0	0.2960805, 0.0021186	0	4.3548	0.0369	0	0	4.3548	0.0369
Sphingobacteriales	0	4.0266949, 0.0288136	0	4.3548	0.0369	0	0	4.3548	0.0369
Coriobacteriales	0.047676, 0.000579	0	0.005974, 0.000093	4.3548	0.0369	3.8571	0.0495	4.3548	0.0369
Cytophagales	0	1.3027542, 0.009322	0	4.3548	0.0369	0	0	4.3548	0.0369
Flavobacteriales	0	0.6513771, 0.00466	0	4.3548	0.0369	0	0	4.3548	0.0369
Caulobacterales	0.005959, 0.000073	0.7994174, 0.0057204	0.001991, 0.000031	3.8571	0.0495	3.8571	0.0495	3.8571	0.0495
Subgroup_17	0	0.0888242, 0.0006356	0	4.3548	0.0369	0	0	4.3548	0.0369
Bifidobacteriales	0.071514, 0.000868	0	0.02987, 0.000466	4.3548	0.0369	3.8571	0.0495	4.3548	0.0369
Spirochaetales	0	0.2960805, 0.0021186	0	4.3548	0.0369	0	0	4.3548	0.0369
Hydrogenophilales	0	0.7994174, 0.0057204	0	4.3548	0.0369	0	0	4.3548	0.0369
Xanthomonadales	0.035757, 0.000435	4.7076801, 0.0336864	0.007965, 0.000124	3.8571	0.0495	3.8571	0.0495	3.8571	0.0495
Alteromonadales	0	0.1776483, 0.0012712	0	4.3548	0.0369	0	0	4.3548	0.0369
Bacillales	0.095351, 0.001158	20.873676, 0.149365	0.039827, 0.000622	3.8571	0.0495	3.8571	0.0495	3.8571	0.0495
Enterobacteriales	4.23718, 0.051464	1.0954979, 0.007839	35.90029, 0.5598	3.8571	0.0495	3.8571	0.0495	3.8571	0.0495
MVP-88	0	0.1184322, 0.000847	0	4.3548	0.0369	0	0	4.3548	0.0369
Rhodospirillales	0	0.2664725, 0.0019068	0	4.3548	0.0369	0	0	4.3548	0.0369
Sphingomonadales	0.005959, 0.000073	0.1184322, 0.000847	0	3.8571	0.0495	4.3548	0.0369	4.3548	0.0369
Mycoplasmatales	0	0	0.053767, 0.000838	0	0	4.3548	0.0369	4.3548	0.0369
Burkholderiales	0.148987, 0.00181	33.960434, 0.243009	0.04381, 0.000684	3.8571	0.0495	3.8571	0.0495	3.8571	0.0495
Pseudomonadales	24.36826, 0.29597	6.9875, 0.05	2.598729, 0.040526	3.8571	0.0495	3.8571	0.0495	3.8571	0.0495
SC-I-84	0	0.0888242, 0.0006356	0	4.3548	0.0369	0	0	4.3548	0.0369
Subgroup_6	0	0.3552966, 0.0025424	0	4.3548	0.0369	0	0	4.3548	0.0369
Neisseriales	0	0.0592161, 0.0004238	0	4.3548	0.0369	0	0	4.3548	0.0369
Lactobacillales	0.143027, 0.001737	1.8060911, 0.0129238	0.007965, 0.000124	3.8571	0.0495	3.8571	0.0495	3.8571	0.0495
Rhizobiales	7.282467, 0.088451	7.1355403, 0.0510594	2.084957, 0.032514	3.8571	0.0495	3.8571	0.0495	3.8571	0.0495
Bacteroidales	0.11323, 0.001375	0	0	4.3548	0.0369	4.3548	0.0369	0	0

Family									
	**Eggs**	**Larvae**	**Adults**	**Eggs vs adults**		**Eggs vs larvae**		**Larvae vs adults**	
**V3-V4**	**Median, IQR[a]**	**Median, IQR[a]**	**Median, IQR[a]**	**Chi-square**	***p*-value**	**Chi-square**	***p*-value**	**Chi-square**	***p*-value**
**Caulobacteraceae**	**0.005959, 0.000073**	**0.7994174, 0.00572**	**0**	**3.8571**	**0.0495**	**4.3548**	**0.0369**	**4.3548**	**0.0369**
**Flavobacteriaceae**	**0**	**0.4441208, 0.003178**	**0**	**4.3548**	**0.0369**	**0**	**0**	**4.3548**	**0.0369**
**Leptospiraceae**	**0**	**0.2960805, 0.002119**	**0**	**4.3548**	**0.0369**	**0**	**0**	**4.3548**	**0.0369**
**Cytophagaceae**	**0**	**1.3027542, 0.00932**	**0**	**4.3548**	**0.0369**	**0**	**0**	**4.3548**	**0.0369**
**Neisseriaceae**	**0**	**0.0592161, 0.0004238**	**0**	**4.3548**	**0.0369**	**0**	**0**	**4.3548**	**0.0369**
**Family_XIII**	**0.017878, 0.000217**	**0**	**0**	**4.3548**	**0.0369**	**4.3548**	**0.0369**	**0**	**0**
**Carnobacteriaceae**	**0.131108, 0.001592**	**0.1480403, 0.001059**	**0**	**3.8571**	**0.0495**	**4.3548**	**0.0369**	**4.3548**	**0.0369**
**Chitinophagaceae**	**0**	**1.9837394, 0.014195**	**0**	**4.3548**	**0.0369**	**0**	**0**	**4.3548**	**0.0369**
**KD3-93**	**0**	**0.1480403, 0.001059**	**0**	**4.3548**	**0.0369**	**0**	**0**	**4.3548**	**0.0369**
**Prevotellaceae**	**0.11323, 0.001375**	**0**	**0**	**4.3548**	**0.0369**	**4.3548**	**0.0369**	**0**	**0**
**Enterobacteriaceae**	**4.23718, 0.051464**	**1.0954979, 0.007839**	**35.9, 0.56**	**3.8571**	**0.0495**	**3.8571**	**0.0495**	**3.8571**	**0.0495**
**Polyangiaceae**	**0**	**0.0888242, 0.0006356**	**0**	**4.3548**	**0.0369**	**0**	**0**	**4.3548**	**0.0369**
**Alcaligenaceae**	**0.089392, 0.001086**	**29.904131, 0.213984**	**0.04, 0**	**3.8571**	**0.0495**	**4.3548**	**0.0369**	**4.3548**	**0.0369**
**Rhizobiaceae**	**0**	**0.2960805, 0.0021186**	**0.01, 0**	**4.3548**	**0.0369**	**5**	**0.0253**	**4.3548**	**0.0369**
**Env.OPS_17**	**0**	**0.2664725, 0.0019068**	**0**	**4.3548**	**0.0369**	**0**	**0**	**4.3548**	**0.0369**
**Lactobacillaceae**	**0**	**1.0658898, 0.0076272**	**0**	**4.3548**	**0.0369**	**0**	**0**	**4.3548**	**0.0369**
**AKYH767**	**0**	**1.0362818, 0.0074152**	**0**	**4.3548**	**0.0369**	**0**	**0**	**4.3548**	**0.0369**
**Pseudomonadaceae**	**24.36826, 0.29597**	**6.5433792, 0.04682**	**2.6, 0.05**	**3.8571**	**0.0495**	**3.8571**	**0.0495**	**3.8571**	**0.0495**
**Mycoplasmataceae**	**0**	**0**	**0.05, 0**	**0**	**0**	**5**	**0.0253**	**5**	**0.0253**
**LiUU-11-161**	**0**	**0.0888242, 0.0006356**	**0**	**4.3548**	**0.0369**	**0**	**0**	**4.3548**	**0.0369**
**Bifidobacteriaceae**	**0.071514, 0.000868**	**0**	**0.03, 0**	**4.3548**	**0.0369**	**4.3548**	**0.0369**	**5**	**0.0253**
**Staphylococcaceae**	**0**	**0.3552966, 0.0025424**	**0**	**4.3548**	**0.0369**	**0**	**0**	**4.3548**	**0.0369**
**Oxalobacteraceae**	**0**	**0.1184322, 0.000847**	**0**	**4.3548**	**0.0369**	**0**	**0**	**4.3548**	**0.0369**
**Moraxellaceae**	**0**	**0.4441208, 0.003178**	**0**	**4.3548**	**0.0369**	**0**	**0**	**4.3548**	**0.0369**
**Hydrogenophilaceae**	**0**	**0.7994174, 0.00572**	**0**	**4.3548**	**0.0369**	**0**	**0**	**4.3548**	**0.0369**
**Rhodocyclaceae**	**0**	**9.6522246, 0.069068**	**0**	**4.3548**	**0.0369**	**0**	**0**	**4.3548**	**0.0369**
**Thermoactinomycetaceae**	**0.023838, 0.000289**	**4.5892479, 0.032839**	**0.02, 0**	**3.8571**	**0.0495**	**4.3548**	**0.0369**	**4.3548**	**0.0369**
**Bacillaceae**	**0.071514, 0.000868**	**15.929131, 0.113984**	**0.02, 0**	**3.8571**	**0.0495**	**4.3548**	**0.0369**	**4.3548**	**0.0369**
**Brucellaceae**	**7.276508, 0.088379**	**4.7076801, 0.033686**	**2.08, 0.03**	**3.8571**	**0.0495**	**3.8571**	**0.0495**	**3.8571**	**0.0495**
**NS9_marine_group**	**0**	**0.2072564, 0.001483**	**0**	**4.3548**	**0.0369**	**0**	**0**	**4.3548**	**0.0369**
**Leuconostocaceae**	**0.011919,**	**0.592161,**	**0**	**3.8571**	**0.0495**	**4.3548**	**0.0369**	**4.3548**	**0.0369**

	0.0001	0.0042							
PHOS-HE51	0	0.0592161, 0.0004238	0	4.3548	0.0369	0	0	4.3548	0.0369
Saprospiraceae	0	0.4441208, 0.003178	0	4.3548	0.0369	0	0	4.3548	0.0369
Burkholderiaceae	0.059595, 0.000724	0.7105932, 0.0050848	0	3.8571	0.0495	4.3548	0.0369	4.3548	0.0369
Bradyrhizobiaceae	0	0.8290254, 0.0059322	0	4.3548	0.0369	0	0	4.3548	0.0369
Comamonadaceae	0	3.2272775, 0.02309	0	4.3548	0.0369	5	0.0253	4.3548	0.0369
Alteromonadaceae	0	0.1776483, 0.0012712	0	4.3548	0.0369	0	0	4.3548	0.0369
Xanthomonadaceae	0.035757, 0.000435	4.4412076, 0.0317796	0.01, 0	3.8571	0.0495	4.3548	0.0369	4.3548	0.0369
Ruminococcaceae	0.351608, 0.004271	0	0.11, 0.01	4.3548	0.0369	3.9706	0.0463	4.5	0.0339
Phyllobacteriaceae	0.005959, 0.000073	1.3027542, 0.009322	0	3.8571	0.0495	4.3548	0.0369	4.3548	0.0369
Sphingomonadaceae	0.005959, 0.000073	0.1184322, 0.000847	0	3.8571	0.0495	4.3548	0.0369	4.3548	0.0369
Rhodospirillaceae	0	0.2664725, 0.0019068	0	4.3548	0.0369	0	0	4.3548	0.0369
Coriobacteriaceae	0.047676, 0.000579	0	0.01, 0	4.3548	0.0369	4.3548	0.0369	5	0.0253

Genus									
	Eggs	Larvae	Adults	Eggs vs adults		Eggs vs larvae		Larvae vs adults	
V3-V4 region	Median, IQR[a]	Median, IQR[a]	Median, IQR[a]	Chi-square	*p*-value	Chi-square	*p*-value	Chi-square	*p*-value
Brevundimonas	**0.005959, 0.000073**	**0.7994174, 0.0057203**	**0.00199, 0.00003**	**3.8571**	**0.0495**	**3.8571**	**0.0495**	**3.8571**	**0.0495**
Trichococcus	**0**	**0.1480403, 0.0010593**	**0**	**4.3548**	**0.0369**	**0**	**0**	**4.3548**	**0.0369**
Turneriella	**0**	**0.2960805, 0.0021186**	**0**	**4.3548**	**0.0369**	**0**	**0**	**4.3548**	**0.0369**
Aeriscardovia	**0.071514, 0.000868**	**0**	**0.0299, 0.0005**	**4.3548**	**0.0369**	**3.8571**	**0.0495**	**4.3548**	**0.0369**
Acinetobacter	**0**	**0.4441208, 0.003178**	**0**	**4.3548**	**0.0369**	**0**	**0**	**4.3548**	**0.0369**
Lentibacillus	**0.023838, 0.000289**	**9.4449682, 0.0675848**	**0.00797, 0.00013**	**3.8571**	**0.0495**	**3.8571**	**0.0495**	**3.8571**	**0.0495**
Ruminococcus	**0.172825, 0.002099**	**0**	**0.0378, 0.0006**	**4.3548**	**0.0369**	**3.8571**	**0.0495**	**4.3548**	**0.0369**
Ralstonia	**0.059595, 0.000724**	**0.6513771, 0.004661**	**0**	**3.8571**	**0.0495**	**4.3548**	**0.0369**	**4.3548**	**0.0369**
Sphingopyxis	**0.005959, 0.000073**	**0.1184322, 0.0008475**	**0**	**3.8571**	**0.0495**	**4.3548**	**0.0369**	**4.3548**	**0.0369**
Dechloromonas	**0**	**1.0362818, 0.0074153**	**0**	**4.3548**	**0.0369**	**0**	**0**	**4.3548**	**0.0369**
Kroppenstedtia	**0.023838, 0.000289**	**4.5892479, 0.032839**	**0.0219, 0.0003**	**3.8571**	**0.0495**	**3.8571**	**0.0495**	**3.8571**	**0.0495**
Atopobium	**0.047676, 0.000579**	**0**	**0.00597, 0.00009**	**4.3548**	**0.0369**	**3.8571**	**0.0495**	**4.3548**	**0.0369**
Flavobacterium	**0**	**0.4441208, 0.003178**	**0**	**4.3548**	**0.0369**	**0**	**0**	**4.3548**	**0.0369**
Leuconostoc	**0.005959, 0.000073**	**0.2072564, 0.001483**	**0.00398, 0.00006**	**3.8571**	**0.0495**	**3.8571**	**0.0495**	**3.8571**	**0.0495**
Sorangium	**0**	**0.0888242, 0.0006**	**0**	**4.3548**	**0.0369**	**0**	**0**	**4.3548**	**0.0369**
Atopostipes	**0.131108, 0.001592**	**0**	**0**	**4.3548**	**0.0369**	**4.3548**	**0.0369**	**0**	**0**
Piscinibacter	**0**	**0.1480403, 0.001059**	**0**	**4.3548**	**0.0369**	**0**	**0**	**4.3548**	**0.0369**
Flavonifractor	**0.005959, 0.000073**	**0**	**0.00597, 0.00009**	**4.3548**	**0.0369**	**0.4286**	**0.5127**	**4.3548**	**0.0369**
Zoogloea	**0**	**0.7402013, 0.0052966**	**0**	**4.3548**	**0.0369**	**0**	**0**	**4.3548**	**0.0369**
Weissella	**0.005959, 0.000073**	**0.3849047, 0.0027542**	**0**	**3.8571**	**0.0495**	**4.3548**	**0.0369**	**4.3548**	**0.0369**
Herbaspirillum	**0**	**0.1184322, 0.0008475**	**0**	**4.3548**	**0.0369**	**0**	**0**	**4.3548**	**0.0369**
Rhizobium	**0**	**0.2960805, 0.0021186**	**0**	**4.3548**	**0.0369**	**4.3548**	**0.0369**	**3.8571**	**0.0495**
Pediococcus	**0**	**0.1184322, 0.0008475**	**0**	**4.3548**	**0.0369**	**0**	**0**	**4.3548**	**0.0369**
Luteibacter	**0.011919, 0.000145**	**0.0592161, 0.0004237**	**0.00398, 0.00006**	**3.8571**	**0.0495**	**3.8571**	**0.0495**	**3.8571**	**0.0495**
Achromobacter	**0.089392, 0.001086**	**29.815307, 0.2133475**	**0.0378, 0.0006**	**3.8571**	**0.0495**	**3.8571**	**0.0495**	**3.8571**	**0.0495**
Thermomonas	**0**	**0.2960805, 0.0021186**	**0**	**4.3548**	**0.0369**	**0**	**0**	**4.3548**	**0.0369**
Azotobacter	**2.294394, 0.027867**	**1.3027542, 0.009322**	**0.175, 0.002**	**3.8571**	**0.0495**	**3.8571**	**0.0495**	**3.8571**	**0.0495**
Intestinimonas	**0.005959, 0.000073**	**0**	**0.00199, 0.00003**	**4.3548**	**0.0369**	**3.8571**	**0.0495**	**4.3548**	**0.0369**
Thauera	**0**	**0.6513771, 0.00466**	**0**	**4.3548**	**0.0369**	**0**	**0**	**4.3548**	**0.0369**
Escherichia-	**4.23718,**	**1.0954979,**	**35.9,**	**3.8571**	**0.0495**	**3.8571**	**0.0495**	**3.8571**	**0.0495**

Shigella	**0.051464**	**0.00784**	**0.5**							
Delftia	**0**	**2.2798199, 0.0163**	**0.00597, 0.00009**	**4.3548**	**0.0369**	**4.3548**	**0.0369**	**3.8571**	**0.0495**
Prevotella	**0.083433, 0.001013**	**0**	**0**	**4.3548**	**0.0369**	**4.3548**	**0.0369**	**0**	**0**
Mycoplasma	**0**	**0**	**0.0538, 0.0008**	**0**	**0**	**4.3548**	**0.0369**	**4.3548**	**0.0369**
Azospirillum	**0**	**0.2664725, 0.0019**	**0**	**4.3548**	**0.0369**	**0**	**0**	**4.3548**	**0.0369**
Lautropia	**0**	**0.0592161, 0.0004237**	**0**	**4.3548**	**0.0369**	**0**	**0**	**4.3548**	**0.0369**
Ferruginibacter	**0**	**0.4441208, 0.003178**	**0**	**4.3548**	**0.0369**	**0**	**0**	**4.3548**	**0.0369**
Dokdonella	**0.011919, 0.000145**	**3.3161017, 0.0237288**	**0**	**3.8571**	**0.0495**	**4.3548**	**0.0369**	**4.3548**	**0.0369**
Oceanobacillus	**0**	**1.6876589, 0.012076**	**0**	**4.3548**	**0.0369**	**0**	**0**	**4.3548**	**0.0369**
BD1-7_clade	**0**	**0.1776483, 0.00127**	**0**	**4.3548**	**0.0369**	**0**	**0**	**4.3548**	**0.0369**
Pseudaminobacter	**0.005959, 0.000073**	**1.3027542, 0.0093**	**0.00199, 0.00003**	**3.8571**	**0.0495**	**3.8571**	**0.0495**	**3.8571**	**0.0495**
Lactobacillus	**0**	**0.9474576, 0.0067797**	**0.00398, 0.00006**	**4.3548**	**0.0369**	**4.3548**	**0.0369**	**3.8571**	**0.0495**
Stenotrophomonas	**0.011919, 0.000145**	**0.6809852, 0.00487**	**0.00398, 0.00006**	**3.8571**	**0.0495**	**3.8571**	**0.0495**	**3.8571**	**0.0495**
Ochrobactrum	**7.276508, 0.088379**	**4.7076801, 0.033686**	**2.08, 0.03**	**3.8571**	**0.0495**	**3.8571**	**0.0495**	**3.8571**	**0.0495**
Candidatus_Accumulibacter	**0**	**1.2139301, 0.008686**	**0**	**4.3548**	**0.0369**	**0**	**0**	**4.3548**	**0.0369**
Bacillus	**0.005959, 0.000073**	**2.8127648, 0.020127**	**0.00398, 0.00006**	**3.8571**	**0.0495**	**3.8571**	**0.0495**	**3.8571**	**0.0495**
Pseudomonas	**22.07386, 0.268**	**5.240625, 0.0375**	**2.42, 0.04**	**3.8571**	**0.0495**	**3.8571**	**0.0495**	**3.8571**	**0.0495**
Staphylococcus	**0**	**0.3552966, 0.0025424**	**0**	**4.3548**	**0.0369**	**0**	**0**	**4.3548**	**0.0369**

[a]Data represent median and interquartile range (IQR) values from pooled populations (i.e from each sheep) of eggs/larvae/adults (male and female) come from three different sheep (natural infection).

Supplementary Table S7: An average composition of significantly different microbial taxa associated with *H. contortus* eggs, larvae and adults using 16S rRNA gene (V5-V7 region). *P*-values were calculated using Kruskal-Wallis test. The level of significance was determined at P < 0.05.

Phylum									
V5-V7 region	Eggs	Larvae	Adults	Eggs vs adults		Eggs vs larvae		Larvae vs adults	
	Median, IQR[a]	Median, IQR[a]	Median, IQR[a]	Chi-square	*p*-value	Chi-square	*p*-value	Chi-square	*p*-value
Bacteroidetes	0.003944, 0.000023	0.72159937, 0.0091729	0.003616, 0.00005	3.8571	0.0495	3.8571	0.0495	3.8571	0.0495
Proteobacteria	33.8843, 0.19932	45.448425, 0.5777345	35.9174, 0.49747	3.8571	0.0495	3.8571	0.0495	3.8571	0.0495
Gemmatimonadetes	0.006574, 0.000038	0.21586306, 0.002744	0.005424, 0.000075	3.8571	0.0495	3.8571	0.0495	3.8571	0.0495
Actinobacteria	0.010518, 0.00006	0.203528, 0.002587	0.012655, 0.000175	3.8571	0.0495	3.8571	0.0495	3.8571	0.0495
Firmicutes	0.084145, 0.000495	0.0801777, 0.0010192	0.047005, 0.000651	3.8571	0.0495	3.8571	0.0495	3.8571	0.0495

Class									
V5-V7	Eggs	Larvae	Adults	Eggs vs adults		Eggs vs larvae		Larvae vs adults	
	Median, IQR[a]	Median, IQR[a]	Median, IQR[a]	Chi-square	*p*-value	Chi-square	*p*-value	Chi-square	*p*-value
Actinobacteria	0.009203, 0.000054	0.203528, 0.002587	0.003616, 0.00005	3.9706	0.0463	3.8571	0.0495	3.9706	0.0463
Gammaproteobacteria	32.58268, 0.19167	7.333177, 0.093219	34.90498, 0.48345	3.9706	0.0463	3.8571	0.0495	3.9706	0.0463
Betaproteobacteria	0.649497, 0.00382	32.83586, 0.4174	0.305534, 0.004232	3.9706	0.0463	3.8571	0.0495	3.9706	0.0463
Coriobacteriia	0.001315, 0.000008	0	0.009039, 0.000126	4.3548	0.0369	3.8571	0.0495	4.3548	0.0369
Clostridia	0.009203, 0.000054	0	0.030734, 0.000425	4.3548	0.0369	3.8571	0.0495	4.3548	0.0369
Bacilli	0.074942, 0.00044	0.080178, 0.00102	0.016271, 0.000225	3.9706	0.0463	3.8571	0.0495	3.9706	0.0463
Gemmatimonadetes	0.006574, 0.000038	0.215863, 0.002744	0.005424, 0.000075	3.9706	0.0463	3.8571	0.0495	3.9706	0.0463
Alphaproteobacteria	0.652127, 0.003836	5.279394, 0.06711	0.706886, 0.00979	3.9706	0.0463	3.8571	0.0495	3.9706	0.0463
Sphingobacteriia	0.003944, 0.000023	0.721599, 0.009173	0.003616, 0.00005	3.9706	0.0463	3.8571	0.0495	3.9706	0.0463

Order									
	Eggs	Larvae	Adults	Eggs vs adults		Eggs vs larvae		Larvae vs adults	
V5-V7 region	Median, IQR[a]	Median, IQR[a]	Median, IQR[a]	Chi-square	*p*-value	Chi-square	*p*-value	Chi-square	*p*-value
Rhodospirillales	0.003944, 0.000023	0.117183, 0.00149	0	3.9706	0.0463	4.3548	0.0369	4.5	0.0339
Enterobacteriales	0.678422, 0.00399	0.567411, 0.007212	31.52242, 0.4366	3.9706	0.0463	3.8571	0.0495	3.9706	0.0463
Coriobacteriales	0.001315, 0.000008	0	0.009039, 0.000126	4.3548	0.0369	3.8571	0.0495	4.3548	0.0369
Xanthomonadales	0.040758, 0.00024	1.332183, 0.016934	0.001808, 0.000025	3.9706	0.0463	3.8571	0.0495	3.9706	0.0463
Micrococcales	0.009203, 0.000054	0.17269, 0.002195	0.001808, 0.000025	3.9706	0.0463	3.8571	0.0495	3.9706	0.0463
Sphingobacteriales	0.003944, 0.000023	0.721599, 0.00917	0.003616, 0.00005	3.9706	0.0463	3.8571	0.0495	3.9706	0.0463
Sphingomonadales	0.005259, 0.00003	0.166523, 0.002117	0	3.9706	0.0463	4.3548	0.0369	4.5	0.0339
Caulobacterales	0.007889, 0.000047	0.604417, 0.007684	0.010847, 0.00015	3.9706	0.0463	3.8571	0.0495	3.9706	0.0463
Clostridiales	0.009203, 0.000054	0	0.030734, 0.000425	4.3548	0.0369	3.8571	0.0495	4.3548	0.0369
Bacillales	0.074942, 0.00044	0.080178, 0.00102	0.016271, 0.000225	3.9706	0.0463	3.8571	0.0495	3.9706	0.0463
Corynebacteriales	0	0.030838, 0.000392	0.001808, 0.000025	4.5	0.0339	4.3548	0.0369	3.9706	0.0463
Burkholderiales	0.649497, 0.00382	32.83586, 0.4174	0.305534, 0.004232	3.9706	0.0463	3.8571	0.0495	3.9706	0.0463
Rhizobiales	0.635035, 0.003736	4.391271, 0.05582	0.696039, 0.009641	3.9706	0.0463	3.8571	0.0495	3.9706	0.0463
Gemmatimonadales	0.006574, 0.000038	0.215863, 0.002744	0.005424, 0.000075	3.9706	0.0463	3.8571	0.0495	3.9706	0.0463
Pseudomonadales	31.8635, 0.18743	5.433582, 0.069071	3.359065, 0.046524	3.9706	0.0463	3.8571	0.0495	3.9706	0.0463

Family									
	Eggs	Larvae	Adults	Eggs vs adults		Eggs vs larvae		Larvae vs adults	
V5-V7 region	Median, IQR[a]	Median, IQR[a]	Median, IQR[a]	Chi-square	*p*-value	Chi-square	*p*-value	Chi-square	*p*-value
Mycobacteriaceae	0	0.030838, 0.000392	0.001810, 0.00002	4.5	0.0339	4.5	0.0339	4.0909	0.0431
Gemmatimonadaceae	0.006574, 0.000038	0.215863, 0.002744	0.005420, 0.00008	3.9706	0.0463	3.8571	0.0495	3.9706	0.0463
Bradyrhizobiaceae	0.002630, 0.000015	0.468731, 0.005958	0.001810, 0.00002	3.9706	0.0463	3.9706	0.0463	4.0909	0.0431
Pseudomonadaceae	31.863496, 0.187432	5.433582, 0.069071	3.360000, 0.05	3.9706	0.0463	3.8571	0.0495	3.9706	0.0463
Enterobacteriaceae	0.678422, 0.00399	0.567412, 0.007213	31.5, 0.4	3.9706	0.0463	3.8571	0.0495	3.9706	0.0463
Coriobacteriaceae	0.001315, 0.000008	0	0.00904, 0.00012	4.3548	0.0369	3.8571	0.0495	4.3548	0.0369
Caulobacteraceae	0.007889, 0.000047	0.604417, 0.007684	0.0108, 0.0002	3.9706	0.0463	3.8571	0.0495	3.9706	0.0463
Hyphomicrobiaceae	0.00263, 0.000015	0.203528, 0.002587	0	3.9706	0.0463	4.3548	0.0369	4.5	0.0339
Chitinophagaceae	0.00263, 0.000015	0.672259, 0.008545	0.00362, 0.00005	3.9706	0.0463	3.8571	0.0495	3.9706	0.0463
Rhodospirillaceae	0.003944, 0.000023	0.117183, 0.00149	0	3.9706	0.0463	4.3548	0.0369	4.5	0.0339
Staphylococcaceae	0.003944, 0.000023	0.018503, 0.000236	0.00542, 0.00008	3.9706	0.0463	3.8571	0.0495	3.9706	0.0463
Comamonadaceae	0.011833, 0.00007	0.339213, 0.004312	0.00181, 0.00002	3.9706	0.0463	3.9706	0.0463	4.0909	0.0431
Burkholderiaceae	0.011833, 0.00007	0.450229, 0.005724	0.00542, 0.00008	3.9706	0.0463	3.8571	0.0495	3.9706	0.0463
Sphingomonadaceae	0.005259, 0.00003	0.166523, 0.002117	0	3.9706	0.0463	4.3548	0.0369	4.5	0.0339
Microbacteriaceae	0.009203, 0.000055	0.172690, 0.002195	0.00181, 0.00002	3.9706	0.0463	3.9706	0.0463	4.0909	0.0431
Xanthomonadaceae	0.040758, 0.00024	1.332184, 0.016935	0.0235, 0.0004	3.9706	0.0463	3.8571	0.0495	3.9706	0.0463
Ruminococcaceae	0.009203, 0.000055	0	0.0307, 0.0004	4.3548	0.0369	3.8571	0.0495	4.3548	0.0369
Phyllobacteriaceae	0.015777, 0.000093	1.005305, 0.012779	0.0145, 0.0002	3.9706	0.0463	3.9706	0.0463	4.0909	0.0431
Rhizobiaceae	0.005259, 0.00003	0.02467, 0.000313	0.00362, 0.00005	3.9706	0.0463	3.8571	0.0495	3.9706	0.0463
Sphingobacteriaceae	0.001315, 0.000008	0.04934, 0.000627	0	3.9706	0.0463	4.3548	0.0369	4.5	0.0339
Brucellaceae	0.60348, 0.00355	2.430001, 0.0309	0.674, 0.009	3.9706	0.0463	3.8571	0.0495	3.9706	0.0463
Alcaligenaceae	0.625831, 0.003681	32.046413, 0.407369	0.298, 0.004	3.9706	0.0463	3.8571	0.0495	3.9706	0.0463
Paenibacillaceae	0.070998, 0.000418	0.061675, 0.000784	0.0108, 0.0002	3.9706	0.0463	3.8571	0.0495	3.9706	0.0463
Rhodobiaceae	0.001315, 0.000008	0.203528, 0.002587	0	3.9706	0.0463	4.3548	0.0369	4.5	0.0339

Genus									
	Eggs	Larvae	Adults	Eggs vs adults		Eggs vs larvae		Larvae vs adults	
V5-V7	Median, IQR[a]	Median, IQR[a]	Median, IQR[a]	Chi-square	*p*-value	Chi-square	*p*-value	Chi-square	*p*-value
Sphingobacterium	0.00131, 0.000009	0.0493400, 0.000627	0	4.0909	0.0431	1	0.3173	4.5	0.0339
Ruminococcus	0.00394, 0.000026	0	0.0090390, 0.000126	1	0.3173	3.9706	0.0463	4.3548	0.0369
Pseudomonas	31.9, 0.157212	5.433582, 0.069071	3.3590650, 0.046524	3.9706	0.0463	3.8571	0.0495	3.9706	0.0463
Ralstonia	0.0118, 0.000068	0.450229, 0.005724	0.005424, 0.000075	4.0909	0.0431	3.9706	0.0463	3.9706	0.0463
Atopobium	0.00131, 0.000009	0	0.009039, 0.0001256	1	0.3173	3.9706	0.0463	4.3548	0.0369
Azospirillum	0.00394, 0.000026	0.117183, 0.00149	0	4.0909	0.0431	1	0.3173	4.5	0.0339
Gemmatimonas	0.00657, 0.000043	0.215863, 0.00274	0.005424, 0.000075	4.0909	0.0431	3.9706	0.0463	3.9706	0.0463
Sphingopyxis	0.00526, 0.000035	0.154188, 0.00196	0	4.0909	0.0431	4.5	0.0339	4.5	0.0339
Parvibaculum	0.00131, 0.000009	0.203528, 0.002587	0	4.0909	0.0431	1	0.3173	4.5	0.0339
GKS98_freshwater_group	0.626, 0.003672	32.046413, 0.407369	0.298302, 0.004132	3.9706	0.0463	3.8571	0.0495	3.9706	0.0463
Paenibacillus	0.071, 0.000406	0.061675, 0.000784	0.010847, 0.00015	4.0909	0.0431	3.9706	0.0463	3.9706	0.0463
Devosia	0.00263, 0.000017	0.178858, 0.002274	0	4.0909	0.0431	1	0.3173	4.5	0.0339
Staphylococcus	0.00394, 0.000026	0.018503, 0.000236	0.005424, 0.000075	4.0909	0.0431	3.9706	0.0463	3.9706	0.0463
Microbacterium	0.0092, 0.00005	0.160355, 0.002038	0.001808, 0.000025	4.0909	0.0431	3.9706	0.0463	3.9706	0.0463
Bosea	0.00131, 0.000009	0.037005, 0.00047	0	4.0909	0.0431	1	0.3173	4.5	0.0339
Brevundimonas	0.00789, 0.000042	0.604417, 0.007684	0.010847, 0.00015	4.0909	0.0431	3.9706	0.0463	3.9706	0.0463
Aquamicrobium	0	0.086345, 0.001097	0.001808, 0.000025	4.5	0.0339	4.3548	0.0369	3.9706	0.0463
Rhizobium	0.00526, 0.000035	0.02467, 0.000314	0.003616, 0.00005	4.0909	0.0431	3.9706	0.0463	3.9706	0.0463
Afipia	0	0.12335, 0.001568	0	4.5	0.0339	0	0	4.5	0.0339
Mycobacterium	0	0.030838, 0.000392	0.001808, 0.000025	4.5	0.0339	4.3548	0.0369	3.9706	0.0463
Escherichia-Shigella	0.678, .004418	0.567411, 0.007212	31.522416, 0.436599	4.0909	0.0431	3.9706	0.0463	3.9706	0.0463
Lysinimonas	0	0.012335, 0.000157	0	4.5	0.0339	0	0	4.5	0.0339
Rhodopseudomonas	0.00131, 0.000009	0.308376, 0.00392	0.001808, 0.000025	4.0909	0.0431	3.9706	0.0463	3.9706	0.0463
Dokdonella	0.0197, 0.00008	0.647589, 0.008232	0.005424, 0.000075	4.0909	0.0431	3.9706	0.0463	3.9706	0.0463
Sphingomonas	0	0.012335, 0.000157	0	4.5	0.0339	0	0	4.5	0.0339
Hyphomicrobium	0	0.02467, 0.000314	0	4.5	0.0339	0	0	4.5	0.0339
Pseudaminobacter	0.0158, 0.000124	0.91896, 0.011682	0.012655, 0.000175	4.0909	0.0431	3.9706	0.0463	3.9706	0.0463

[a]Data represent median and interquartile range (IQR) values from pooled populations (i.e. from each sheep) of eggs/larvae/adults (male and female) come from three different sheep (natural infection).

Supplementary Table S8: Microbial community composition associated with the different life-cycle stages of *H. contortus* (V3-V4 region)

Genus	*H. contortus* eggs[a]	*H. contortus* larvae[a]	*H. contortus* adults[a]
Brevundimonas	**0.0000368552±0.005951424**	**0.8008475±0.003116797**	**1.990000e-003±0.0000156179**
Trichococcus	**0**	**0.1483051±0.000577185**	**0**
Turneriella	**0**	**0.2966102±0.001154369**	**0**
Aeriscardovia	**0.000442262±0.07141708**	**0**	**0.0298±0.000234269**
Acinetobacter	**0**	**0.4449153±0.001731554**	**0**
Lentibacillus	**0.000147421±0.02380569**	**9.461864±0.03682439**	**0.00796±0.0000624716**
Ruminococcus	**0.001068801±0.1725913**	**0**	**0.0378±0.00029674**
Ralstonia	**0.000368552±0.05951424**	**0.6525424±0.002539613**	**0**
Sphingopyxis	**0.0000368552±0.005951424**	**0.1186441±0.000461748**	**0**
Dechloromonas	**0**	**1.038136±0.004040293**	**0**
Kroppenstedtia	**0.000147421±0.02380569**	**4.597457±0.01789273**	**0.0219±0.000171797**
Atopobium	**0.000294842±0.04761139**	**0**	**0.00597±0.0000468537**
Flavobacterium	**0**	**0.4449153±0.001731554**	**0**
Leuconostoc	**0.0000368552±0.005951424**	**0.2076271±0.000808059**	**3.980000e-003±0.0000312358**
Sorangium	**0**	**0.08898305±0.000346311**	**0**
Atopostipes	**0.000810814±0.1309313**	**0**	**0**
Piscinibacter	**0**	**0.1483051±0.000577185**	**0**
Flavonifractor	**0.0000368552±0.005951424**	**0**	**0.00597±0.0000468537**
Zoogloea	**0**	**0.7415254±0.002885924**	**0**
Weissella	**0.0000368552±0.005951424**	**0.3855932±0.00150068**	**0**
Herbaspirillum	**0**	**0.1186441±0.000461748**	**0**
Rhizobium	**0**	**0.2966102±0.001154369**	**0.00597±0.0000468537**
Pediococcus	**0**	**0.1186441±0.000461748**	**0**
Luteibacter	**0.0000737104±0.01190285**	**0.05932203±0.000230874**	**3.980000e-003±0.0000312358**
Achromobacter	**0.000552828±0.08927135**	**29.868640±0.116245**	**0.0378±0.00029674**
Thermomonas	**0**	**0.2966102±0.001154369**	**0**
Azotobacter	**0.01418925±2.291298**	**1.305085±0.005079225**	**0.175±0.001374376**
Intestinimonas	**0.0000368552±0.005951424**	**0**	**1.990000e-003±0.0000156179**
Thauera	**0**	**0.6525424±0.002539613**	**0**
Escherichia-Shigella	**0.02620405±4.231462**	**1.097458±0.004271167**	**35.90±0.2815597**
Delftia		**2.283898±0.008888645**	**0.00597±0.0000468537**
Prevotella	**0.000515973±0.08331993**	**0**	**0**
Mycoplasma	**0**	**0**	**0.0537±0.000421684**
Azospirillum	**0**	**0.2669491±0.001038932**	**0**
Lautropia	**0**	**0.05932203±0.000230874**	**0**
Ferruginibacter	**0**	**0.4449153±0.001731554**	**0**
Dokdonella	**0.0000737104±0.01190285**	**3.322034±0.01292894**	**0**
Oceanobacillus	**0**	**1.690678±0.006579906**	**0**
BD1-7_clade	**0**	**0.1779661±0.000692622**	**0**
Pseudaminobacter	**0.0000368552±0.005951424**	**1.305085±0.005079225**	**1.990000e-003±0.0000156179**
Lactobacillus	**0**	**0.9491525±0.003693982**	**3.980000e-003±0.0000312358**
Stenotrophomonas	**0.0000737104±0.01190285**	**0.6822034±0.00265505**	**3.980000e-003±0.0000312358**

Ochrobactrum	**0.0450002±7.266688**	**4.716102±0.01835447**	**2.07±0.01627386**
Candidatus_Accumulibacter	**0**	**1.216102±0.004732915**	**0**
Bacillus	**0.0000368552±0.005951424**	**2.817797±0.01096651**	**3.980000e-003±0.0000312358**
Pseudomonas	**0.1365117±22.044070**	**5.250000±0.02043234**	**2.42±0.019007**
Staphylococcus	**0**	**0.3559322±0.001385243**	**0**

[a]Data represent median and interquartile range (IQR) values from pooled populations (i.e from each sheep) of eggs/larvae/adults (male and female) come from three different sheep ± standard deviation (SD).

Supplementary Table S9: Microbial community composition associated with the different life-cycle stages of *H. contortus* (V5-V7 region) isolated from ovine hosts at the genus

Genus	*H. contortus* Eggs[a]	*H. contortus* Larvae[a]	*H. contortus* Adults[a]
Sphingobacterium	1.310000e-003±3.866980e-006	0.04913106±0.000362117	0
Ruminococcus	3.940000e-003±0.0000116009	0	0.009014423±0.0000662498
Pseudomonas	31.90±0.09371617	5.410558±0.03987813	3.349760±0.02461842
Ralstonia	0.0118±0.0000348028	0.4483209±0.003304317	0.005408654±0.0000397499
Atopobium	1.310000e-003±0.00000386698	0	0.009014423±0.0000662498
Azospirillum	3.940000e-003±0.0000116009	0.1166863±0.000860028	0
Gemmatimonas	0.00657±0.0000193349	0.2149484±0.001584262	0.005408654±0.0000397499
Sphingopyxis	0.00526±0.0000154679	0.1535346±0.001131616	0
Parvibaculum	1.310000e-003±0.00000386698	0.2026656±0.001493733	0
GKS98_freshwater_group	0.626±0.001840681	31.910620±0.235195	0.297476±0.002186243
Paenibacillus	0.071±0.000208817	0.06141382±0.000452646	0.01081731±0.0000794997
Devosia	2.630000e-003±0.00000773395	0.1781001±0.001312674	
Staphylococcus	3.940000e-003±0.0000116009	0.01842415±0.000135794	0.005408654±0.0000397499
Microbacterium	0.0092±0.0000270688	0.1596759±0.00117688	0.001802885±0.00001325
Bosea	1.310000e-003±0.00000386698	0.0368483±0.000271588	0
Brevundimonas	0.00789±0.0000232019	0.6018555±0.004435933	0.01081731±0.0000794997
Aquamicrobium	0	0.08597936±0.000633705	0.001802885±0.00001325
Rhizobium	0.00526±0.0000154679	0.02456553±0.000181058	0.003605769±0.0000264999
Afipia	0	0.1228276±0.000905292	0
Mycobacterium	0	0.03070691±0.000226323	0.001802885±0.00001325
Escherichia-Shigella	0.678±0.00199536	0.5650072±0.004164345	31.435100±0.2310262
Lysinimonas	0	0.01228276±0.0000905292	0
Rhodopseudomonas	1.310000e-003±0.00000386698	0.3070691±0.002263231	0.001802885±0.00001325
Dokdonella	0.0197±0.0000580046	0.6448452±0.004752785	0.005408654±0.0000397499
Sphingomonas	0	0.01228276±0.0000905292	0
Hyphomicrobium	0	0.02456553±0.000181058	0
Pseudaminobacter	0.0158±0.0000464037	0.915066±0.006744429	0.01262019±0.0000927497

[a]Data represent average values from pooled populations (i.e from each sheep) of eggs/larvae/adults (male and female) come from three different sheep± standard deviation (SD).

Supplementary Table S10: Microbial community composition associated with the adult stage of *H. contortus* at population (clusters of male and female worms) and individual (single adult female worm) levels (V3-V4 region) isolated from ovine hosts at the genus level.

**Genus**	***H. contortus* adults (population level)(Average, ±SD)[a]**	***H. contortus* adults (Individual female worm level)(Average, ±SD)[b]**
Brevundimonas	**1.990000e-003±0.0000156179**	**0.00163131 ± 0.00015272**
Trichococcus	**0**	**0**
Turneriella	**0**	**0**
Aeriscardovia	**0.0298±0.000234269**	**0.02447166 ± 0.00228877**
Lentibacillus	**0.00796±0.0000624716**	**0.004894266 ± 0.000458**
Ruminococcus	**0.0378±0.00029674**	**0.19414332 ± 0.01815669**
Ralstonia	**0**	**0**
Sphingopyxis	**0**	**0**
Dechloromonas	**0**	**0**
Kroppenstedtia	**0.0219±0.000171797**	**0.00815722 ± 0.00076303**
Atopobium	**0.00597±0.0000468537**	**0.004894266 ± 0.0004577**
Flavobacterium	**0**	**0**
Sorangium	**0**	**0**
Atopostipes	**0**	**0**
Piscinibacter	**0**	**0**
Flavonifractor	**0.00597±0.0000468537**	**0.004894266 ± 0.0004577**
Zoogloea	**0**	**0**
Weissella	**0**	**0**
Herbaspirillum	**0**	**0**
Rhizobium	**0.00597±0.0000468537**	**0.00163131 ± 0.00015272**
Pediococcus	**0**	**0**
Luteibacter	**3.980000e-003±0.0000312358**	**0.003262955 ± 0.000305**
Achromobacter	**0.0378±0.00029674**	**0.02773495 ± .002593656**
Thermomonas	**0**	**0**
Intestinimonas	**1.990000e-003±0.0000156179**	**0.00163131 ± 0.00015272**
Thauera	**0**	**0**
Escherichia-Shigella	**35.90±0.2815597**	**29.4119116 ± 2.75065773**
Delftia	**0.00597±0.0000468537**	**0.003262955 ± 0.000305**
Prevotella	**0**	
Mycoplasma	**0.0537±0.000421684**	**0.044049058 ± 0.0041198**
Azospirillum	**0**	**0**
Lautropia	**0**	**0**
Ferruginibacter	**0**	**0**
Dokdonella	**0**	**0**
Oceanobacillus	**0**	**0**
BD1-7_clade	**0**	**0**
Pseudaminobacter	**1.990000e-003±0.0000156179**	**0.00163131 ± 0.00015272**
Lactobacillus	**3.980000e-003±0.0000312358**	**0.00163131 ± 0.00015272**

Stenotrophomonas	3.980000e-003±0.0000312358	0.004894266 ± 0.0004577
Ochrobactrum	2.07±0.01627386	1.699978513 ± 0.1589851
Candidatus_Accumulibacter	0	0
Bacillus	3.980000e-003±0.0000312358	0.00163131 ± 0.00015272
Pseudomonas	2.42±0.019007	1.16812319 ± 0.10924532
Leuconostoc	3.980000e-003±0.0000312358	0
Azotobacter	0.175±0.001374376	0
Acinetobacter	0	0.004894266 ± 0.0004577
Staphylococcus	0	0.00652591 ± 0.00061032
Sphingomonas	0	0.00163131 ± 0.00015272
Aquamicrobium	0	0.00163131 ± 0.00015272
RC9_gut_group	0	0.003262955 ± 0.000305
Shinella	0	0.003262955 ± 0.000305

[a]Data represent average values from pooled populations (i.e from each sheep) of adults (clusters of male and female) come from three different sheep± standard deviation (SD).

[b]Data represent average values from individual single female worms (i.e from each sheep) come from three different sheep± standard deviation (SD).
